# Magnesium modulates the stress responses of oral streptococci to environmental and antibiotic challenges by altering cell envelope and nutrient transport pathways

**DOI:** 10.3389/fmicb.2025.1669039

**Published:** 2026-01-06

**Authors:** Surabhi Mishra

**Affiliations:** Department of Biomedical and Applied Sciences, Indiana University School of Dentistry Indianapolis, Indianapolis, IN, United States

**Keywords:** magnesium, oral streptococci, *Streptococcus mutans*, efflux pump, HlyX

## Abstract

**Background:**

Magnesium (Mg^2+^) is one of the most abundant metals in human teeth, second only to calcium. Demineralization of the tooth, caused by sugar intake or acid reflux, releases Mg^2+^ into the saliva. Mg^2+^ is also recommended as a dietary supplement for the prevention and treatment of chronic diseases. Oral streptococci, therefore, must regulate Mg^2+^ homeostasis to adapt to fluctuating levels of saliva in the human oral cavity.

**Materials and methods:**

We determined the toxic concentration of MgCl_2_ for *Streptococcus* spp. and used a sub-toxic dose to assess its effect on osmotic and cation-excess stress tolerance. Growth assays, ICP-MS, proteomic analysis, and lipidomic analysis were performed on wild-type and mutant strains lacking a putative Mg^2+^ efflux pump homolog.

**Results:**

Mg^2+^ supplementation enhanced tolerance to osmotic and cation-excess stress in both caries-associated and commensal streptococci. Homologs of the magnesium protection factor A (MpfA) were found across *Streptococcus* groups. Mutants lacking *mpfA* homologs (*smu_1693* in *S. mutans*, and *ssa1761* in *S. sanguinis*) showed MgCl_2_ sensitivity. Despite unchanged intracellular Mg^2+^ levels in Δ*smu_1693*, the mutant exhibited stress tolerance, consistent with the disruption of magnesium efflux pumps. Proteomic and lipidomic analyses revealed altered levels of amino acid transporters, cell envelope proteins, and an increase in long-chain unsaturated fatty acids. Furthermore, modulating intracellular Mg^2+^ concentration, either by MgCl_2_ supplementation or by eliminating HlyX, impacted the efficacy of multiple cell wall-targeting antibiotics.

**Conclusion:**

This study highlights the role of Mg^2+^ in enhancing stress tolerance and modulating antibiotic sensitivity in streptococci, using *S. mutans* as a model.

## Introduction

The human oral cavity harbors over 700 species of bacteria, in addition to fungi, archaea, viruses, and protists, collectively comprising the human oral microbiome (Chen et al., 2010; Ghannoum et al., 2010; Belmok et al., 2020; Pride et al., 2012; Abeles et al., 2014; Wantland et al., 1958; Redanz et al., 2020). Reflecting the diversity in composition, various sites within the oral cavity create a heterogeneous environment that supports microbial colonization, particularly with surface hardness, oxygen levels, and nutrient availability, which favor the microbes residing in specific niches within the oral cavity. One significant group of bacteria isolated from diverse oral sites includes members of the genus *Streptococcus*, represented by approximately 18 species within the oral microbiome (Baty et al., 2022). This genus comprises early tooth colonizers, as well as commensals and pathogens (Griffen et al., 2012; Richards et al., 2017). The successful colonization of various oral sites suggests that streptococci can effectively withstand environmental stressors, including changes in pH, temperature, oxygen, and nutrient availability.

Metals are key nutrients that influence bacterial metabolism, biofilm formation, and interactions with the host (Remick and Helmann, 2023). Only a select few metals are utilized by living organisms for successful growth and survival, with magnesium (Mg^2+^) being essential and the second most abundant (Remick and Helmann, 2023; Romani and Scarpa, 2000). Typical cellular concentrations of Mg^2+^ in both prokaryotes and eukaryores are in the millimolar (mM) range (15–25 mM) (Maguire and Cowan, 2002; Romani and Scarpa, 1992; Moomaw and Maguire, 2008), where it plays critical roles, including stabilizing ribosomes, neutralizing the charge on anionic molecules such as DNA and RNA, and acting as a cofactor for various metabolic enzymes, particularly those utilizing nucleotides (reviewed in Remick and Helmann, 2023; de Baaij et al., 2015). Beyond supporting routine growth and survival, Mg^2+^ transport and homeostasis are vital to the virulence attributes of pathogenic bacteria (Papp-Wallace and Maguire, 2008; Garcia Vescovi et al., 1996; Martini et al., 2020; Derzelle et al., 2004). Phagocytic cells utilize Mg^2+^ deprivation as an effective mechanism to kill bacteria after engulfment, highlighting a strict requirement for Mg^2+^ in bacterial growth and survival (Papp-Wallace and Maguire, 2008). Although Mn^2+^, K^+^, and Fe^2+^ can partially substitute for Mg^2+^ in functions like ribosome stabilization, their distinct coordination chemistry, bio-availability, and reactivity prevent them from entirely replacing Mg^2+^ in other essential cellular processes (Remick and Helmann, 2023; Nierhaus, 2014). The critical requirement of Mg^2+^ for bacterial growth and virulence suggests that bacteria must possess mechanisms to obtain Mg^2+^ from their environment and maintain physiologically relevant levels within the cytoplasm.

The stringent requirement for Mg^2+^ extends to humans, where most is stored in bones and teeth alongside calcium and phosphorus (Aziz et al., 2018; Gilfrich et al., 1981). Although tooth minerals are not directly accessible to bacteria, acids—from diet, gastroesophageal reflux disease (GERD) (Ranjitkar et al., 2012), or microbial metabolism (Loesche, 1986)—can lower salivary pH, leading to demineralization and Mg^2+^ release into saliva (Abou Neel et al., 2016). Saliva, a key nutrient source for oral microbes, contains Mg^2+^ levels that fluctuate in response to dietary and supplement intake. Mg^2+^ is commonly added to the diets to reduce the risk of type II diabetes (Dong et al., 2011; Hruby et al., 2014) and cardiovascular disease (Tangvoraphonkchai and Davenport, 2018). In addition to being a key Mg^2+^ source for oral streptococci, fluctuations in salivary Mg^2+^ levels influence interspecies interactions (Cheng et al., 2020). Commensal streptococci like *Streptococcus gordonii* and *Streptococcus sanguinis* promote H_2_O_2_ production in an Mg^2+^-dependent manner, inhibiting the growth of cariogenic *Streptococcus mutans* (Cheng et al., 2020). This antagonism supports the idea that Mg^2+^ may be a potential prebiotic for oral health. Low dietary Mg^2+^ intake has been linked to oral dysbiosis-related diseases such as periodontitis and dental caries (Rajesh et al., 2015), prompting recommendations for Mg^2+^ supplementation alongside Ca^2+^ and phosphorus to reduce caries risk (Meisel et al., 2016). Given the dynamic nature of Mg^2+^ levels in the oral cavity, understanding Mg^2+^ transport and homeostasis in oral streptococci is essential.

Mg^2+^ transporters have been identified in model bacteria like *Salmonella enterica*, *Escherichia coli*, *Bacillus subtilis*, and *Staphylococcus aureus* (Papp-Wallace and Maguire, 2008; Hmiel et al., 1989; Kehres and Maguire, 2002; Wakeman et al., 2014; Armitano et al., 2016), and fall into four families: CorA, MgtE, MgtA/B, and CorC/HlyC. CorA is an important Mg^2+^ transporter with orthologues present in almost every phylum, while another Mg^2+^ channel protein, MgtE is found in bacteria and archaea (reviewed in Franken et al., 2022). MgtA/B belongs to a broader family of P-type ATPase metal transporters involved in the transport of cations, including Ca^2+^, Mg^2+^, and transition metals, with very high sequence and structure similarity (reviewed in Franken et al., 2022). The role of CorC/HlyC family of proteins in Mg^2+^ efflux was proposed as early as the 1990s (Gibson et al., 1991); however, their physiological significance was recognized only recently when spontaneous mutants of *S. aureus* and *B. subtilis*, lacking a functional homolog of CorC/HlyC, failed to grow under cold or Mn^2+^-excess stress, respectively (Armitano et al., 2016; Pi et al., 2020).

This study evaluated the response of exogenous Mg^2+^ supplementation on the growth and stress tolerance ability of *S. mutans* and other streptococci. In general, supplementation with moderate doses of MgCl_2_ was found to improve streptococcal tolerance to osmotic stress and divalent metal toxicity. All streptococcal genomes examined in this work contain a homolog of Mg^2+^ efflux pump, annotated as HlyX. *S. mutans* and *S. sanguinis ΔhlyX* strains showed an increase in sensitivity to high doses of Mg^2+^; however, *S. mutans ΔhlyX* did not accumulate Mg^2+^ as observed in other bacteria. The proteome and lipidome of untreated and MgCl_2_-treated *S. mutans* wild-type and *ΔhlyX* strains were analyzed to understand the protective mechanism of MgCl_2_.

## Materials and methods

### Bacterial strains, plasmids, and growth conditions

Bacterial strains and plasmids used in this study are listed in Supplementary Table S1. *Streptococcus* spp. belonging to mutans, mitis, salivarius, pyogenic, sanguinis, and downei groups were maintained in brain heart infusion (BHI) medium at 37 °C in a 5% CO_2_/95% air atmosphere (v/v). *S. mutans* strain UA159 (Ajdić et al., 2002) was used as the wild-type strain, and all the *S. mutans* strains were derived from this strain. *S. mutans strains* were routinely grown in Todd-Hewitt broth (BBL, Becton Dickinson) supplemented with 0.3% yeast extract (THYE) at 37 °C in a 5% CO_2_/95% air atmosphere (v/v). The antibiotic concentrations utilized included 1 mg mL^−1^ of spectinomycin, 10 μg mL^−1^ of chloramphenicol, and 10 μg mL^−1^ of erythromycin. The *E. coli* DH10B strains harboring the plasmids were cultivated in Luria Bertani (LB) medium supplemented with 25 μg mL^−1^ of chloramphenicol.

### Growth and efficiency of plating assays

Growth curves of *S. mutans* wild-type and mutant strains were generated using a Bioscreen C system (Oy Growth Curves AB Ltd., Helsinki, Finland). Overnight cultures were diluted 1:50 in fresh THYE medium, grown to mid-exponential phase (OD₆₀₀ ≈ 0.4), and further diluted 1:100 into THYE with or without MgCl_2_ supplementation in microtiter plate wells. Wells were overlaid with sterile mineral oil to maintain anaerobic conditions. OD_600_ readings were recorded every 30 min following 10 s of shaking.

For the EOP assay, OD_600_ of overnight cultures in THYE were adjusted to 0.2 in fresh THYE, followed by 10-fold serial dilutions (10^−1^ to 10^−6^). A 4 μL of each dilution was spotted on THYE/BHI agar plates with or without metal supplements. For the pH 5.2 agar plates, the medium pH was adjusted using 1 N HCl. Plates were air-dried for 30 min to dry the spots and then incubated at 37 °C for 2 days in a 5% CO_2_/95% air atmosphere. Images were taken.

### Construction of strains and plasmids

Deletion mutants of *hlyX* and *rpmH* were generated via allelic replacement using antibiotic resistance markers. Replacement constructs were assembled using the NEB HiFi DNA Assembly Kit by fusing PCR-amplified resistance genes (*aad9* or *ermB*) with ~700 bp flanking regions of the target genes. These constructs were introduced into *S. mutans* UA159 via transformation facilitated by competence-stimulating peptide CSP-18. Transformants were verified by PCR and confirmed by sequencing. For complementation, the Δ*hlyX* mutant was transformed with plasmid pIB166 carrying the *hlyX* gene inserted between *BamHI* and *HindIII* sites.

### Measurement of intracellular metal ions by ICP-MS

Intracellular metal content in *S. mutans* wild-type and mutant strains was quantified using inductively coupled plasma mass spectrometry (ICP-MS) at the UF-IFAS Analytical Services Laboratories. Overnight cultures were grown in THYE medium with appropriate antibiotics, diluted 1:100 into fresh THYE (4 × 30 mL replicates), and incubated to mid-log phase (OD_600_ ≈ 0.4). Cells were harvested (4 °C, 15 min, 4,000 rpm), resuspended in THYE with or without 20 mM MgCl_2_, and incubated for 1.5 h. After a second harvest, the cells were washed with PBS containing 0.2 mM EDTA, followed by a wash with PBS. Pellets were resuspended in 35% HNO_3_ and heated at 95 °C for 1 h, then diluted to 3.5% HNO_3_ with metal-free water. Metal concentrations were measured using an Agilent 7900 ICP-MS and normalized to total protein content determined by the BCA assay (Pierce).

### Sample preparation for proteomic and lipidomic analysis

Overnight grown wild-type and mutant strains in THYE medium were diluted 1:50 in 10 mL THYE and grown till mid-log phase. Mid-log phase cultures were diluted 1:100 in 250 mL THYE and grown until the OD at 600 nm reached ~0.4. Cells were centrifuged and resuspended in fresh THYE with or without 20 mM MgCl_2_ and incubated for 1.5 h at 37 °C in a 5% CO_2_ atmosphere. Cells were centrifuged at 4 °C for 15 min at 3,600 rpm and then washed once with 0.02 M PBS. Pellets were frozen in dry ice and sent on dry ice for proteomic and lipidomic analysis to Creative Biogen Inc. (NY).

### Proteomic analysis

Cell pellets were lysed in buffer (2% SDS, Tris-HCl pH 8, 1% PMSF) by sonication. Debris was removed by centrifugation (20,000 × g, 15 min, 4 °C), and protein concentration was determined using the BCA assay. Equal amounts of protein were used per sample. Disulfide bridges were reduced by 10 mM Tris(2-carboxyethyl) phosphine (TCEP) at 56 °C for 1 h, followed by alkylation with 20 mM iodoacetamide (IAA) in the dark at room temperature for 30 min. Proteins were precipitated overnight with six volumes of pre-chilled (−20 °C) acetone and reconstituted in 250 μL of 100 mM triethylammonium bicarbonate (TEAB) buffer. Digestion was performed overnight at 37 °C using trypsin (1:50, w/w, Promega). Peptides (1 μg) were analyzed using an Ultimate 3000 nano UHPLC system (Thermo Scientific, Waltham, MA) equipped with a PepMap C18 trapping nanocolumn (100 Å, 100 μm × 2 cm, 5 μm) and an analytical column (PepMap C18, 100 Å, 75 μm × 50 cm, 2 μm). The mobile phase consisted of A: 0.1% formic acid in water; B: 0.1% formic acid in acetonitrile, and the flow rate was maintained at 250 nL/min. The gradient was as follows: 2–8% buffer B over 3 min, 8–22% over 39 min, 22–36% over 8 min, and 36–90% over 4 min. The mobile phases were: A—0.1% formic acid in water; B—0.1% formic acid in acetonitrile. The flow rate was maintained at 250 nL/min. Mass spectrometry was performed with a full scan range of 300–1,650 *m*/*z* at a resolution of 60,000 (at 200 *m*/*z*), with an AGC target of 3 × 10^6^. MS/MS scans were acquired in top 20 mode using the following parameters: resolution 15,000 (at 200 *m*/*z*), AGC target 1 × 10^5^, maximum injection time 19 ms, normalized collision energy 28%, isolation window 1.4 Th, charge state exclusion (unassigned, 1, >6), and dynamic exclusion of 30 s. Raw MS files were analyzed using MaxQuant (v1.6.2.14) against the *Streptococcus mutans* protein database. Search parameters included: fixed modification—carbamidomethylation (C); variable modification—oxidation (M); enzyme specificity—trypsin; precursor mass tolerance—10 ppm; MS/MS tolerance—0.5 Da. Only high-confidence peptides were retained for downstream protein identification.

### Bioinformatic analysis of the proteomic data

Approximately 1,500 proteins were identified per analysis. Proteins with fold-change ratios >1.2 or <0.83 (1/1.2) were considered significantly altered. Bioinformatics analyses were performed to characterize these quantifiable proteins, including Cluster of Orthologous Groups (COG), Gene Ontology (GO), and KEGG pathway annotation.

#### GO annotation and GO enrichment analysis

Gene Ontology (GO) provides a unified framework for describing gene and protein functions across species, encompassing three domains: biological process (BP), molecular function (MF), and cellular component (CC). In this study, GO annotations were derived from the UniProt-GOA database.^1^ Identified protein IDs were first converted to UniProt IDs and then mapped to GO terms. Proteins were categorized into BP, MF, and CC domains.

Enrichment analysis was performed using Fisher’s exact test to compare differentially expressed proteins against the background of all identified proteins. GO terms with a corrected *p*-value <0.05 were considered significantly enriched. The GO hierarchy, structured as a directed acyclic graph (DAG), was further analyzed using the R package topGO to explore functional relationships among enriched terms.

#### KEGG annotation and KEGG pathway enrichment analysis

The Kyoto Encyclopedia of Genes and Genomes (KEGG) integrates molecular interaction networks (Pathway database), gene and protein information (Gene database), and biochemical compounds and reactions (Compound and Reaction databases), forming interconnected “protein” and “chemical” networks. KEGG pathway enrichment analysis was performed using Fisher’s exact test to compare differentially expressed proteins against the background of all identified proteins. Pathways with a corrected *p*-value <0.05 were considered significantly enriched. Protein annotations were conducted using KOBAS 3.0, a widely used tool for KEGG-based pathway and disease enrichment analysis.

#### COG annotation and cluster analysis

The Clusters of Orthologous Groups (COG) database, established in 1997, provides functional annotation across 26 categories and includes data from 62 genomes (46 bacterial, 13 archaeal, and 3 eukaryotic). Proteins were considered significantly differentially abundant if they met the criteria: |log₂(fold change)| >log₂(1.2) and *p* < 0.05, with *p*-values calculated using a two-tailed *t*-test. Protein expression patterns across sample groups were analyzed by hierarchical clustering. Expression values were transformed using the function *x* = −log₂(*X*), and clustering was performed using Euclidean distance and average linkage in Genesis software. Cluster membership was visualized as a heatmap using the heatmap.2 function from the gplots R package.

### Untargeted lipidomic analysis

Cell pellets were thawed on ice and resuspended in 1.5 mL of a 2:1 (v/v) chloroform: methanol solution. The mixture was then vortexed for 1 min, followed by the addition of 0.5 mL of ultrapure water. Cells were lysed by sonication for 30 min at 4 °C. Lipids were extracted by centrifugation (3,000 rpm, 10 min, 4 °C), and the lower organic phase was collected and dried under nitrogen. Dried extracts were resuspended in 200 μL of a 1:1 (v/v) isopropanol: methanol solution, and 5 μL of lysophosphatidic acid (LPC 12:0, 0.14 mg/mL) was added as an internal standard. After centrifugation (12,000 rpm, 10 min, 4 °C), supernatants were analyzed by UPLC-MS. Lipid separation was performed using an ACQUITY UPLC BEH C18 column (100 × 2.1 mm, 1.7 μm) on a UPLC system coupled to a Q Exactive MS (Thermo Scientific). The mobile phases were: A—60% acetonitrile, 40% H_2_O, 10 mM ammonium formate; B—10% acetonitrile, 90% isopropanol, 10 mM ammonium formate. The gradient elution was: 0–1.0 min, 30% B; 1.0–10.5 min, 30–100% B; 10.5–12.5 min, 100–30% B; 12.51–16 min, 30% B. Flow rate was 0.3 mL/min; column temperature was 40 °C; sample manager temperature was 4 °C.

Mass spectrometry parameters in ESI positive and ESI negative modes were as follows:

*ESI positive mode*: Heater temp 300 °C; sheath gas 45 arb; auxiliary gas 15 arb; sweep gas 1 arb; spray voltage 3.0 kV; capillary temp 350 °C; S-lens RF level 30%.
*ESI negative mode*: Same as above, except spray voltage 3.2 kV and S-lens RF level 60%.

### Statistical analysis

Raw data were processed using LipidSearch (Thermo) for peak alignment based on *m*/*z* and retention time. Positive and negative ion mode data were merged and analyzed using SIMCA-P (v14.1). Principal component analysis (PCA) was used for unsupervised data visualization and outlier detection. Supervised models, including partial least squares discriminant analysis (PLS-DA) and orthogonal PLS-DA (OPLS-DA), were used to identify potential biomarkers. Biomarkers were filtered based on VIP >1.5, fold change >2, and *p* < 0.05 (*t*-test). QC samples were run at regular intervals in both ion modes to monitor system stability. Ion features from QC samples were used to calculate relative standard deviation (RSD), with most features showing RSD <30%, indicating robust system performance.

### Etest strip assay

Etest strips (bioMérieux) were used to determine the minimum inhibitory concentrations (MICs) of various antibiotics. Overnight *S. mutans* cultures were diluted 1:50 in THYE medium and grown to mid-log phase. A 100 μL aliquot of each culture was mixed with 0.7% THYE top agar and spread evenly onto THYE agar plates. After solidification (30 min), antibiotic strips were placed at the center of each plate. Plates were incubated at 37 °C for 24 h in a 5% CO_2_ atmosphere, and MIC values were recorded.

## Results

### Oral streptococci exhibit reduced tolerance to exogenous Mg^2+^ supplementation compared to pyogenic streptococci

**Figure 1:**
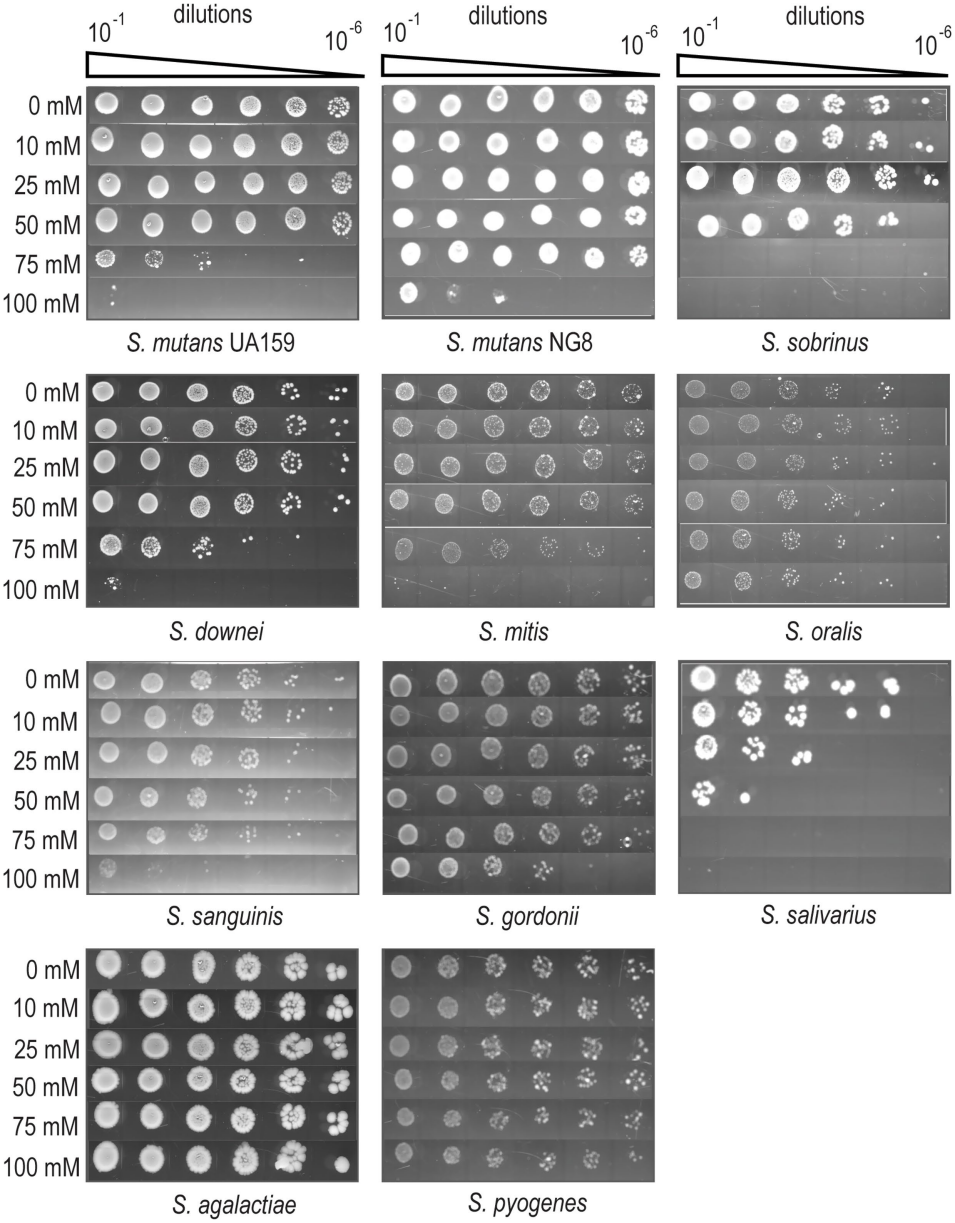
Effect of exogenous MgCl_2_ supplementation on the growth of *Streptococcus* spp. The EOP assay demonstrates the growth of *Streptococcus* spp. on BHI supplemented with 0, 10, 25, 50, 75, and 100 mM MgCl_2_. Overnight cultures grown in BHI were adjusted to an OD_600_ of 0.2 and serially diluted 10-fold. A 4 μL aliquot from each dilution (10^−1^ to 10^−6^) was spotted onto BHI agar plates. Plates were incubated at 37 °C in a 5% CO_2_ atmosphere for 2 days. The images shown are representative of four independent experiments.

To assess Mg^2+^ homeostasis in oral streptococci, we first determined the range of Mg^2+^ concentrations tolerated by representative *Streptococcus* species using an efficiency of plating (EOP) assay. Brain-heart infusion (BHI) agar supplemented with increasing concentrations of MgCl_2_ (0–100 mM) was employed for this analysis. This assay included representative species from major oral streptococcal groups (mitis, sanguinis, salivarius, downei, mutans) and two pyogenic species (*S. agalactiae* and *S. pyogenes*), which are not typically found in the oral cavity (Gaballa et al., 2003). BHI medium, containing ~0.6 mM Mg^2+^ (measured by ICP-MS), supported the growth of all tested species. All oral streptococcal species showed mild to moderate growth inhibition at ≥75 mM MgCl_2_, with *S. salivarius* and *S. sobrinus* being most sensitive, and *S. oralis* and *S. gordonii* being the most tolerant (Figure 1). In contrast, the pyogenic streptococci *S. agalactiae* and *S. pyogenes* displayed robust growth even at 100 mM MgCl_2_, indicating a significantly higher tolerance to exogenous Mg^2+^ compared to oral streptococci (Figure 1). This observation is consistent with the physiological environments these species typically inhabit, as human serum contains higher Mg^2+^ concentrations (~1.7–1.9 mM) than saliva (~0.5 mM) (Meisel et al., 2016; Singhal et al., 2023). These dose-dependent effects helped define the optimal Mg^2+^ concentrations for downstream studies on Mg^2+^ homeostasis.

### Exogenous Mg^2+^ supplementation confers protection to streptococci against metal toxicity and osmotic stress

Previous studies in *B. subtilis* have demonstrated that an increase in intracellular Mg^2+^ concentration mitigates transition metal toxicity and osmotolerance (Pi et al., 2020; Wendel et al., 2022). Therefore, we hypothesized that elevated salivary Mg^2+^ levels may enhance the tolerance of oral streptococci to environmental stressors. To test this hypothesis, we screened representative oral streptococci for stress tolerance in BHI supplemented with 20 mM MgCl_2_. Stress conditions included acid stress (pH 5.2), osmotic shock (0.3 M KCl, 0.5 M NaCl), and exposure to transition metals (5 mM FeSO_4_, 5 mM MnCl_2_, 3 mM ZnCl_2_, 2 mM CoCl_2_). Stressor concentrations were based on prior work with *S. mutans* and adjusted for more sensitive species as needed (Mishra and Brady, 2021).

**Figure 2:**
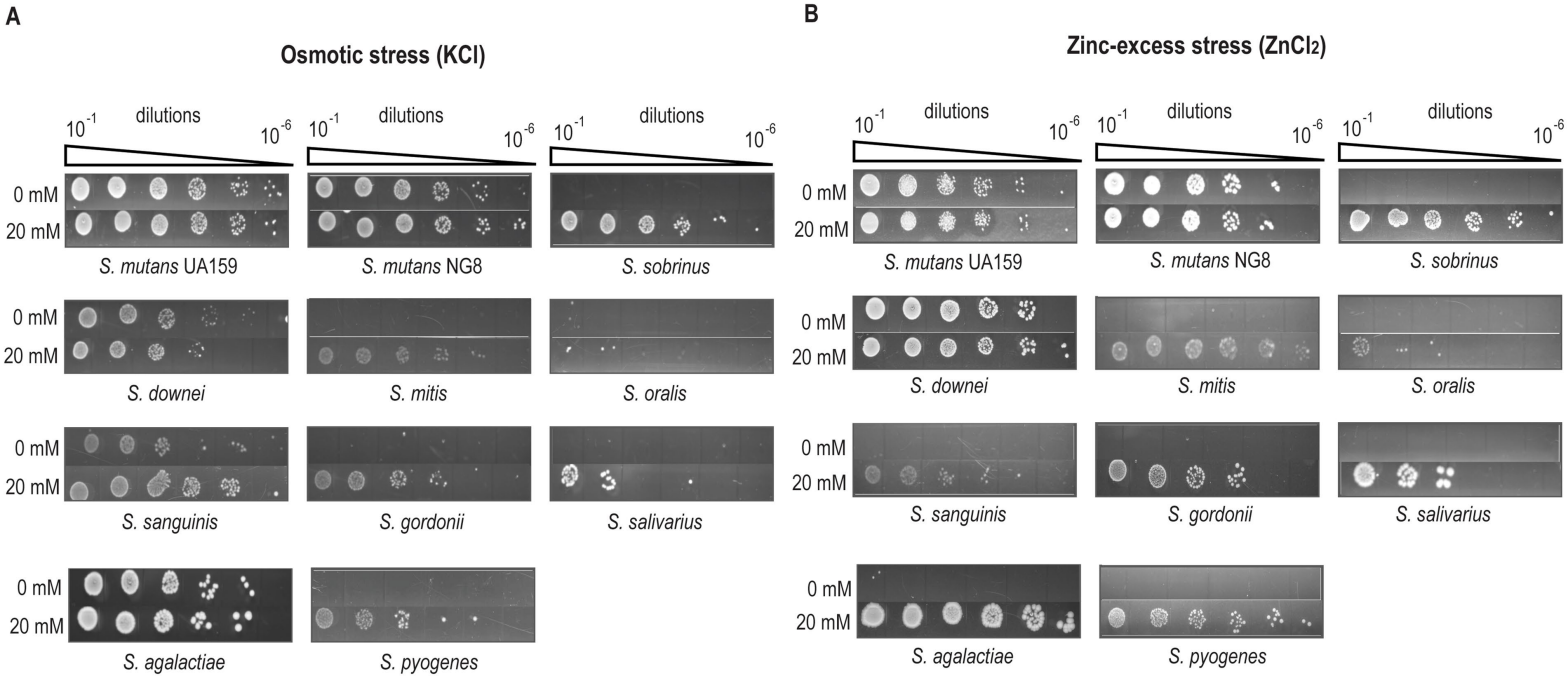
MgCl_2_ supplementation promotes the survival of various *Streptococcus* spp. when exposed to **(A)** osmotic stress (0.3 M KCl), and **(B)** Zn-toxicity (2.5 mM ZnCl_2_). Overnight cultures of *Streptococcus* spp. were diluted in BHI to an OD_600_ of 0.2, followed by 10-fold serial dilutions. A 4 μL aliquot from each dilution (10^−1^ to 10^−6^) was spotted onto BHI agar plates containing either 0.3 M KCl ± 20 mM MgCl_2_ or 2.5 mM ZnCl_2_ ± 20 mM MgCl_2_. Plates were incubated at 37 °C in a 5% CO_2_ atmosphere for 2 days. Images shown are representative of three independent experiments.

*Streptococcus* spp. exhibited variable growth under acid stress (Supplementary Figure S1), with *S. mutans*, *S. downei*, and *S. agalactiae* displaying greater resistance to low pH compared to the moderately sensitive *S. mitis* and *S. gordonii* (Supplementary Figure S1). The higher acid resistance of *S. gordonii* compared to the related *S. sanguinis* has been reported in the literature (Cheng et al., 2020). A distinct Mg^2+^-dependent protection against acid stress was clearly observed in the cariogenic *S. sobrinus*, while other acid-sensitive species showed no such response (Supplementary Figure S1). The lack of Mg^2+^-mediated protection in the mitis and sanguinis groups can be attributed to the presence of SpxB, a Mg^2+^ −dependent hydrogen peroxide (H_2_O_2_)-producing pyruvate oxidase (Cheng et al., 2020; Cheng et al., 2018), which is absent in *S. sobrinus*. The oxidative stress induced by SpxB likely counteracts the protective effects of Mg^2+^. These findings suggest that under acidic and Mg^2+^-rich conditions, the competitive balance may shift in favor of SpxB-deficient, acid-sensitive species like *S. sobrinus*, thereby mimicking the demineralization environment of the oral cavity. Similar to their response under acid stress, various *Streptococcus* species exhibited differential tolerance to KCl-induced osmotic stress (Figure 2A). *S. mutans*, *S. downei*, *S. sanguinis*, and *S. agalactiae* showed greater resistance to 0.3 M KCl, while other species were more sensitive. However, supplementation with MgCl_2_ significantly rescued the growth of all *Streptococcus* species that were adversely affected by KCl-induced osmotic stress (Figure 2A), suggesting a protective role of magnesium ions under KCl-induced osmotic challenge. Sensitivity to osmotic stress remained largely unchanged across *Streptococcus* spp. when osmolyte was changed from KCl to NaCl, with the exception of *S. pyogenes*, which showed high tolerance to NaCl (Supplementary Figure S2). This response is not surprising, as both Na^+^ and K^+^ though carry single positive charge, vary in reactivity, size, and abundance, likely contributing to distinct physiological roles (Remick and Helmann, 2023). Unlike the consistent Mg^2+^-dependent protection observed under KCl-induced stress, the protective effect of Mg^2+^ under NaCl stress was less evident, with only *S. gordonii* showing improved growth (Supplementary Figure S2). In fact, *S. downei* and *S. sanguinis* exhibited increased sensitivity to NaCl-induced osmotic challenge in the presence of MgCl₂. Meanwhile, *S. mutans* and pyogenic streptococci tolerated NaCl well, whereas *mitis* streptococci and *S. salivarius* were more sensitive. These differences underscore the distinct physiological roles of K^+^ and Na^+^ in bacterial systems (Remick and Helmann, 2023). Notably, adaptation to K^+^-induced osmotic stress involves rapid efflux of Mg^2+^ followed by reuptake during recovery, highlighting the importance of K^+^/Mg^2+^ balance in cellular homeostasis (Wendel et al., 2022).

We next assessed whether MgCl_2_ supplementation could protect streptococci from toxicity caused by biologically relevant transition metal-excess (Fe^2+^, Mn^2+^, Co^2+^, and Zn^2+^). Most species showed minimal sensitivity to Fe^2+^, Mn^2+^, and Co^2+^ at tested concentrations (Supplementary Figures S3–S5). Notably, the concentrations of these metal salts tested represent the maximum levels that can be visibly detected in a growth assay without compromising visibility and fall within the range tested for other bacteria (Pi et al., 2020; Guan et al., 2015). As previously reported, *S. mutans* exhibited high Zn^2+^ tolerance (Ganguly et al., 2021), while no other *Streptococcus* spp., except *S. downei*, showed tolerance to Zn^2+^-excess (Figure 2B). Mg^2+^ supplementation conferred protection against Zn^2+^ toxicity in all species except, *S. mutans* and *S. downei*, which were inherently tolerant to high Zn^2+^ (Figure 2B). These findings support a protective role for Mg^2+^ against both osmotic and metal-induced stress. Further validation will require analysis of Mg^2+^ transporter mutants, particularly those affecting Mg^2+^ efflux.

### *In silico* identification of putative Mg^2+^ efflux pumps in streptococcal genomes

Mg^2+^ efflux functions for CorB, CorC, and CorD were first reported in *S. typhimurium* in 1991 (Gibson et al., 1991). Decades later, a Mg^2+^ protection factor (MpfA) was identified in *S. aureus* and *B. subtilis*, supporting growth under moderate Mg^2+^ levels (up to 10 mM) (Armitano et al., 2016; Pi et al., 2020). A paralog, MpfB, was later discovered in *S. aureus* (Trachsel et al., 2019), differing from MpfA by a shorter C-terminal region. Using Basic Local Alignment Search Tool (BLAST) (Johnson et al., 2008), we identified a single MpfA/B homolog in all representative streptococcal genomes (Table 1), each 443–457 amino acids in length and resembling MpfA due to their extended C-termini. A multiple sequence alignment illustrating the conservation of residues among HlyX homologs from *Streptococcus* spp. and *Bacillus subtilis* MpfA as well as *S. aureus* MpfB is shown in Supplementary Figure S6. Bacterial MpfA/B and CorB/C/D family proteins possess a conserved pair of cystathionine-beta-synthase (CBS) domains (Gaballa et al., 2003). The streptococcal homologs of MpfA contain both a pair of CBS and C-terminal cyclic nucleotide monophosphate (CNNM) domains and are annotated as hemolysins (HlyX). These HlyX proteins from different streptococcal groups exhibit a significant degree of amino acid sequence identity (~75%) and a similar topology, comprising four transmembrane domains. None of the HlyX homologs have been characterized; however, a platelet-binding function was initially proposed for *S. sanguinis* HlyX (Ssa_1761) (Martini et al., 2020). A hemolysin function has been ruled out for *S. sanguinis* HlyX (Martini et al., 2020). Given the high degree of amino acid sequence similarity among all streptococcal HlyXs, these proteins are more likely involved in Mg^2+^ homeostasis than cytolysis.

**Table 1:** List of genes/proteins pertinent to Mg^2+^ efflux pump in representative oral *Streptococcus* spp.

*Streptococcus* spp.	MpfA/B homologs
*S. mitis*	Smi_0320; CBS domain membrane protein (443 AA, 33%)
*S. oralis* ATCC 49296	HMPREF8578_0158; CBS domain protein (443 AA, 34%)
*S. sanguinis SK36*	SSA_1761; putative hemolysin (446 AA, 32%)
*S. gordonii*	SGO_1655; CBS domain protein/possible hemolysin (446 AA, 32%)
*S. anginosus* strain F0211	HMPREF0813_00712; hemolysin protein family (446 AA, 36%)
*S. salivarius* K12	RSSL_00706; Mg^2+^ and Co^2+^ efflux protein CorC (443 AA, 34%)
*S. thermophilus*	stu1641; predicted membrane protein of the hemolysin family (443 AA, 34%)
*S. downei*	NCTC11391_01919; hemolysins and related proteins containing CBS domains (445 AA, 34%)
*S. criceti* HS-6	STRCR_2137; CBS domain protein (445 AA, 35%)
*S. mutans* str. UA159	SMU_1693; putative hemolysin (445 AA, 34%)
*S. sobrinus* W1703	HMPREF1557_00886; phage tail component protein (457 AA, 33%)
*S. ratti* strain DSM 22768	HHO37_01045; HlyC/CorC family transporter (445 AA, 34%)
*S. pyogenes*	SpyM3_0276; hemolysin family transporter (444 AA, 34%)
*S. agalactiae*	gbs1469; hemolysin (444 AA, 31%)

### HlyX protects *Streptococcus* spp. from Mg^2+^ toxicity

**Figure 3:**
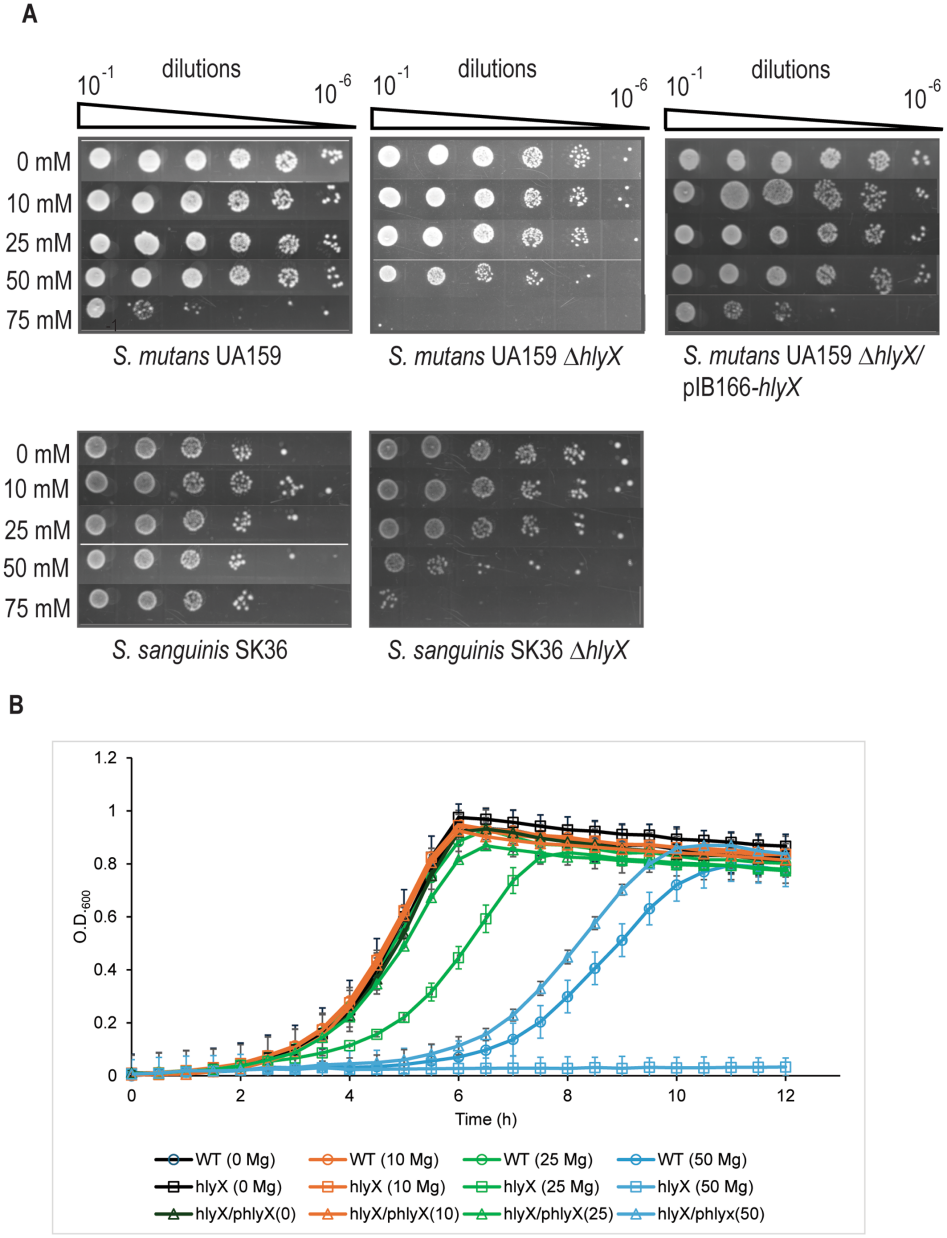
Deletion of *hlyX* enhances the sensitivity of *S. mutans* and *S. sanguinis* to exogenous MgCl_2_ supplementation in the growth medium. **(A)** EOP assay. Overnight cultures of wild-type (*S. mutans* UA159 and *S. sanguinis* SK36), Δ*hlyX* mutants (derived from each parent strain), and the complemented *S. mutans* Δ*hlyX*/pIB166-*hlyX* strain were diluted in their respective media to an OD_600_ of 0.2, followed by 10-fold serial dilutions. A 4 μL aliquot from each dilution (10^−1^ to 10^−6^) was spotted onto media plates containing varying concentrations of MgCl_2_. *S. mutans* strains were grown and assayed in THYE, while BHI was used for *S. sanguinis*. Plates were incubated at 37 °C in a 5% CO_2_ atmosphere for 2 days. Images shown are representative of four independent experiments. **(B)** Growth profile of *S. mutans* wild-type, *ΔhlyX*, and complemented strain in THYE supplemented with 0, 10, 25, and 50 mM MgCl_2_. Overnight cultures were diluted 1:50 in THYE medium and grown to mid-log phase (OD_600_ ≈ 0.4), then diluted 1:100 into fresh THYE containing varying concentrations of MgCl₂. Growth was monitored using a Bioscreen C system in triplicate wells overlaid with mineral oil to maintain anaerobic conditions. For the complemented strain, 10 μg/mL chloramphenicol was added to maintain plasmid selection. Experiments were performed in triplicate, and the data shown represent the mean of triplicates from one representative experiment. Error bars represent standard deviation of three technical replicates from an individual experiment.

Given the presence of CBS and CNNM domains and similarity to MpfA, we hypothesized that HlyX functions as a Mg^2+^ efflux protein in streptococci. To test this, we deleted *hlyX* (*smu_1693*) in *S. mutans* UA159, a genetically tractable model for dental caries research. The Δ*hlyX* mutant showed no growth defect in complex media under standard conditions (Figures 3A,B). However, in the presence of ≥50 mM MgCl_2_, the mutant exhibited impaired growth in both broth and agar. This phenotype was rescued by plasmid-based complementation of *hlyX*, confirming its role in Mg^2+^ tolerance (Figure 3A).

To determine if this function is conserved, we tested a Δ*hlyX* mutant in *S. sanguinis* SK36, which also failed to grow on complex media with 75 mM MgCl_2_, mirroring the *S. mutans* phenotype (Figure 3A). These results suggest that HlyX proteins act as Mg^2+^ efflux pumps across streptococcal species.

### Measurement of intracellular Mg^2+^ levels by ICP-MS

To test whether HlyX functions as an Mg^2+^ efflux pump in *S. mutans*, we measured intracellular Mg^2+^ levels in wild-type and Δ*hlyX* strains using ICP-MS. While wild-type cells maintained stable Mg^2+^ levels even with 20 mM MgCl_2_ supplementation, the Δ*hlyX* mutant showed 50% lower Mg^2+^ levels in unsupplemented medium. Upon MgCl_2_ supplementation, intracellular Mg^2+^ in the mutant doubled, reaching wild-type levels (Table 2). This pattern contrasts with *B. subtilis* Δ*mpfA* mutants, which accumulate Mg^2+^ (Pi et al., 2020), suggesting alternative Mg^2+^ efflux mechanisms in *S. mutans*. Despite reduced Mg^2+^, Δ*hlyX* grew normally in THYE, indicating sufficient Mg^2+^ for basal growth.

**Table 2:** Intracellular metal levels measured by ICP-MS.

Growth conditions	Strains	Mg (mg of metal/mg of protein)	K (mg of metal/mg of protein)	Na (mg of metal/mg of protein)	Fe (ng of metal/mg of protein)	Mn (ng of metal/mg of protein)	Zn (ng of metal/mg of protein)	Cu (ng of metal/mg of protein)	Co (ng of metal/mg of protein)
THYE	UA159	0.63 ± 0.13	4.56 ± 0.69	90.1 ± 13.2	48 ± 3.43	24.0 ± 9.16	95.9 ± 8.2	19.03 ± 2.3	0.11 ± 0.01
THYE	*ΔhlyX*	0.28 ± 0.06*	5.25 ± 0.88	111.1 ± 18.6	51.6 ± 8.71	14.9 ± 2.13	107.8 ± 20.14	17.8 ± 3.02	0.07 ± 0.02*
THYE	*ΔrpmH*	0.30 ± 0.09*	23.02 ± 6.4*	458.7 ± 131*	130.9 ± 13.3*	15.02 ± 1.8*	130.2 ± 12*	37.9 ± 2.9*	ND
THYE + 20 mM MgCl_2_	UA159	0.50 ± 0.05	4.63 ± 0.97	98.16 ± 21.2	49.8 ± 7.84	14.3 ± 2.34*	100.4 ± 16.14	18.9 ± 4.9	0.07 ± 0.01*
THYE + 20 mM MgCl_2_	*ΔhlyX*	0.57 ± 0.09	4.4 ± 0.76	92.5 ± 16.8	47.2 ± 6.22	15.3 ± 2.48	104.6 ± 8.94	17.15 ± 2.7	0.09 ± 0.02
THYE + 20 mM MgCl_2_	*ΔrpmH*	0.56 ± 0.06	21.9 ± 1.32*	433.1 ± 22.7*	141.2 ± 19.6*	17.2 ± 3.6	225.5 ± 28*	73.5 ± 29.4*	0.21 ± 0.05*

Mid-log phase cultures (x4) were incubated in THYE ± 20 mM MgCl_2_ for 1.5 h before harvesting for measurement of intracellular metal ions by ICP-MS. Metal concentrations were normalized to the total protein content (expressed as milligrams of protein). Statistical significance was determined using a two-tailed independent Student’s *t*-test, comparing each treatment group to the untreated UA159 control. A *p*-value less than 0.05 (*) was considered statistically significant.

To further explore Mg^2+^-dependent growth, we analyzed a Δ*rpmH* mutant lacking ribosomal protein L34, known to destabilize ribosomes and reduce intracellular Mg^2+^ in *B. subtilis* (Akanuma et al., 2014). The presence of low intracellular Mg^2+^ led to reduced growth of the *B. subtilis* Δ*rpmH* mutant, which could be partially restored by supplementation with Mg^2+^ salts (Akanuma et al., 2014). The *S. mutans* Δ*rpmH* strain showed reduced growth on THYE, which was restored by MgCl_2_ supplementation (Supplementary Figure S7). ICP-MS revealed that Δ*rpmH* and Δ*hlyX* had comparable Mg^2+^ levels, but Δ*rpmH* also accumulated significantly higher levels of Na^+^, K^+^, Fe^2+^, and Cu^2+^, even with Mg^2+^ supplementation (Table 2). Collectively, intracellular Mg^2+^ levels failed to explain the growth phenotype of the *S. mutans* Δ*rpmH* strain. It is plausible that other cations in the Δ*hlyX* strain took over Mg^2+^-mediated functions to sustain growth. For instance, K^+^ often substitutes for Mg^2+^ in ribosomal structures, facilitating growth. To investigate whether HlyX influences the levels of other metal ions, the concentrations of Na^+^, K^+^, Fe^2+^, Mn^2+^, Co^2+^, Cu^2+^, and Zn^2+^ were quantified in both wild-type and Δ*hlyX* strains (Table 2). The ICP-MS results did not reveal any significant differences in the concentrations of these metals between the wild-type and Δ*hlyX* strains (Table 2), supporting the hypothesis that metal homeostasis was maintained in both strains, regardless of the exogenous MgCl_2_ (20 mM) supplementation. However, in contrast to the Δ*hlyX* strain, the intracellular concentrations of Na^+^, K^+^, Fe^2+^, and Cu^2+^ were several-fold higher in the Δ*rpmH* strain (Table 2). Notably, the intracellular concentration was not restored to the wild-type or the Δ*hlyX* strain levels, even when Mg^2+^ levels were replenished through exogenous MgCl_2_ supplementation (Table 2). Additionally, the levels of Zn^2+^, Cu^2+^, and Co^2+^ increased with MgCl_2_ supplementation, whereas the intracellular concentration of Mn^2+^ remained unaffected under all testing conditions in the Δ*rpmH* strain (Table 2). Thus, the ICP-MS analysis of the Δ*rpmH* mutant suggests that the release of free Mg^2+^ attributable to structural perturbations of the ribosomes, rather than the total Mg^2+^ pool, influenced the intracellular metal pool and subsequently affected the growth of the mutant strain in *S. mutans*. Also, the increase in Mg^2+^ levels in MgCl_2_-supplemented Δ*rpmH* likely prevented the mis-metalation of Mg^2+^-cofactored enzymes and proteins, restoring growth.

### *ΔhlyX* strain protects from osmotic stress and Co^2+^ toxicity

**Figure 4:**
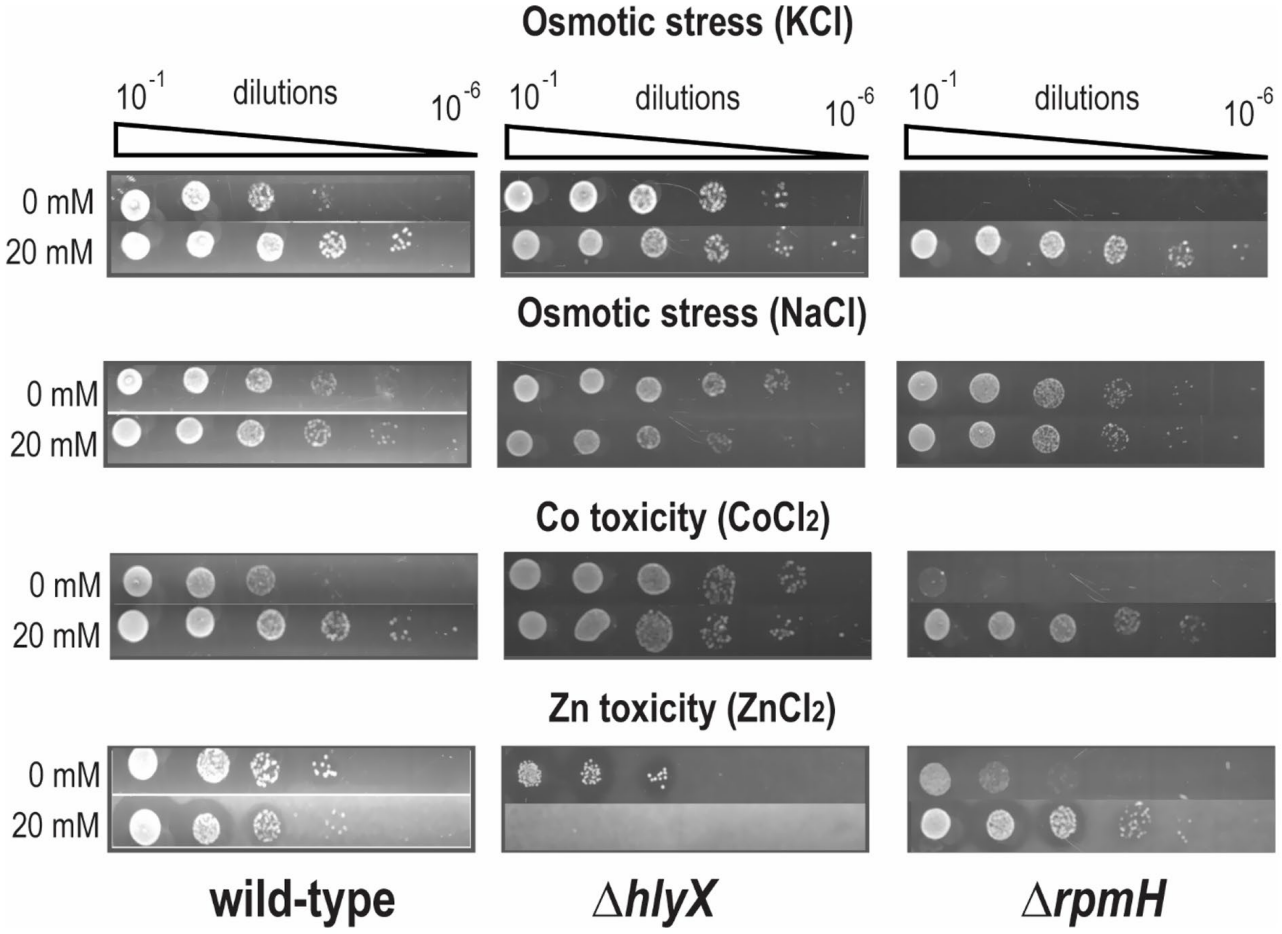
MgCl_2_ supplementation protects wild-type, *ΔhlyX*, and *ΔrpmH* strains against osmotic stress and metal toxicity. Overnight cultures of wild-type and mutant strains were diluted in THYE to an OD_600_ of 0.2, followed by 10-fold serial dilutions. A 4 μL aliquot from each dilution (10^−1^ to 10^−6^) was spotted onto THYE agar plates containing various environmental stressors: 0.3 M KCl, 0.5 M NaCl, 2 mM CoCl_2_, and 4 mM ZnCl_2_. Plates were incubated at 37 °C in a 5% CO₂ atmosphere for 2 days. Images shown are representative of three independent experiments.

Studies in *B. subtilis* have shown that Mg^2+^ accumulation supports osmoadaptation and resistance to metal toxicity. As ICP-MS analysis revealed no increase in intracellular Mg^2+^ in the *S. mutans* Δ*hlyX* mutant, unlike *B. subtilis*, we compared the growth of wild-type, Δ*hlyX*, and Δ*rpmH* strains under osmotic and metal stress. Under KCl stress, Δ*hlyX* outperformed the wild-type, while Δ*rpmH* failed to grow. MgCl_2_ supplementation improved growth in all strains, confirming its generally protective function (Figure 4). ICP-MS showed that Δ*rpmH* accumulated fivefold more K^+^, likely contributing to its growth defect under KCl-induced stress. In contrast, NaCl stress had minimal impact (Figure 4), and Mg^2+^ supplementation did not improve growth—suggesting independent roles for Na^+^ and Mg^2+^.

We next assessed Mg^2+^-mediated protection against divalent metal toxicity. High concentrations of Fe^2+^ and Mn^2+^ did not inhibit growth, and precipitation of the salts in the media limited assay reliability. Therefore, we focused on Co^2+^ and Zn^2+^. Under Co^2+^ stress, Δ*hlyX* showed enhanced tolerance, further improved by Mg^2+^, similar to *B. subtilis* Δ*mpfA*. However, Δ*hlyX* was more sensitive to Zn^2+^, and Mg^2+^ worsened this effect, indicating a role for HlyX in Zn^2+^ detoxification (Figure 4). In contrast, Mg^2+^ alleviated Zn^2+^ toxicity in Δ*rpmH*, but not in wild-type or Δ*hlyX*, suggesting that Zn^2+^/Mg^2+^ imbalance may lead to mis-metalation of metalloproteins. These findings underscore the significance of intracellular Mg^2+^ in stress tolerance and indicate that HlyX plays a crucial role in maintaining metal homeostasis in *S. mutans*.

### Mg^2+^ supplementation enhances the abundance of cell envelope proteins and transporters

**Figure 5:**
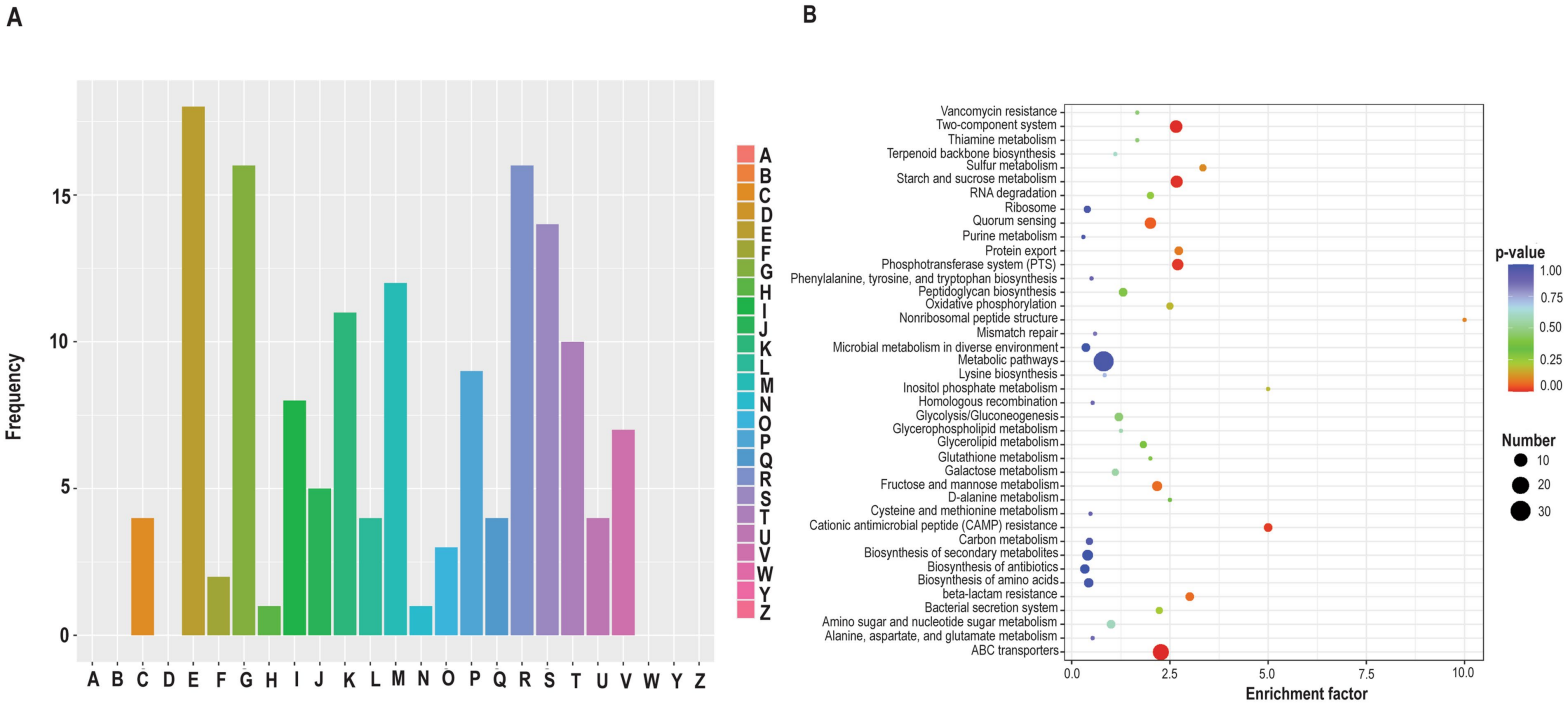
A comparative analysis of the differentially expressed proteins (DEPs) between MgCl_2_-treated and untreated wild-type *S. mutans* cultures. Proteins were considered significantly differentially expressed if they met the criteria: |log_2_(fold change)| >log_2_(1.2) and *p* < 0.05, with *p*-values calculated using a two-tailed *t*-test method. **(A)** Classification of DEPs annotated in the cluster of orthologous groups (COGs) database (https://www.ncbi.nlm.nih.gov/COG/). The *x*-axis represents COG terms, and the *y*-axis represents protein count in each COG functional class (A–W, Y, Z). A: RNA processing and modification (0); B: Chromatin structure and dynamics (0); C: Energy production and conversion (4); D: Cell cycle control, cell division, chromosome partitioning (0); E: Amino acid transport and metabolism (18); F: Nucleotide transport and metabolism (2); G: Carbohydrate transport and metabolism (16); H: Coenzyme transport and metabolism (1); I: Lipid transport and metabolism (8); J: Translation, ribosome structure, and biogenesis (5); K: Transcription (11); L: Replication, recombination and repair (4); M: Cell wall/membrane/envelope biogenesis (12); N: Cell motility (1); O: Posttranslational modification, protein turnover, chaperones (3); P: Inorganic ion transport and metabolism (9); Q: Secondary metabolites biosynthesis, transport and catabolism (4); R: General function prediction only (16); S: Function unknown (14); T: Signal transduction mechanisms (10); U: Intracellular trafficking, secretion and vesicular transport (4); V: Defense mechanisms (7); W: Extracellular structure (0); Y: Nuclear structure (0); and Z: Cytoskeleton (0). The numbers in parentheses include the number of proteins. **(B)** Kyoto Encyclopedia of Genes and Genomes (KEGG) enrichment analysis of the DEPs. The *x*-axis displays the enrichment factor, and the *y*-axis represents the pathway name. The number of proteins and the −log10(*p*-value) are indicated by the size and color of the dots, respectively. Protein pathway annotation was performed using the KEGG database. KOBAS 3.0, a widely used tool for functional annotation and enrichment analysis, was employed to assign KEGG pathway descriptions to the differentially expressed proteins (DEPs). Enrichment analysis was conducted using Fisher’s exact test to evaluate the significance of DEP enrichment against the background of all identified proteins. Pathways with a corrected *p*-value <0.05 were considered statistically significant. Additionally, DEPs were classified into five major functional classes based on KEGG pathway definitions, excluding those used solely for enrichment analysis.

To better understand the mechanisms underlying Mg^2+^-dependent stress tolerance in *S. mutans*, a comparative proteomic analysis was performed on log-phase wild-type cells grown in THYE medium with or without 20 mM MgCl_2_. Approximately 1,500 proteins were identified, with 173 differentially expressed (127 upregulated, 46 downregulated; Supplementary Table S2). Functional annotation and enrichment analyses—including Gene Ontology (GO), Clusters of Orthologous Groups (COG), and KEGG pathway mapping—were conducted to assess the physiological roles of DEPs. GO up and down and GO enrichment analyses revealed that Mg^2+^ supplementation primarily affected proteins involved in transport (biological process and molecular function) and membrane localization (cellular component) (Supplementary Figures S8, S9A–C). COG analysis further highlighted enrichment in amino acid transport and metabolism (18 proteins), carbohydrate metabolism (16 proteins), cell wall/membrane/envelope biogenesis (12 proteins), transcription (11 proteins), and signal transduction (10 proteins) (Figure 5A). KEGG pathway analysis identified transporters, signaling pathways, and metabolic processes among the top 40 enriched pathways (Figure 5B).

Notable upregulated proteins included those involved in rhamnose-glucose polysaccharide biosynthesis (RgpG, RgpA), cell wall anchoring (WapA, WapE, SpaP), signal transduction (LiaF, Smu_44), metal transport (SloC, AdcA, FeoB, SloA, PacL), and polyamine transport (PotA, PotB) (Supplementary Table S2). Downregulated proteins included the sorbitol transporter and glucosyltransferases GtfC and GtfD.

### HlyX elimination and Mg^2+^ supplementation showed distinct yet overlapping proteomic signatures

**Figure 6:**
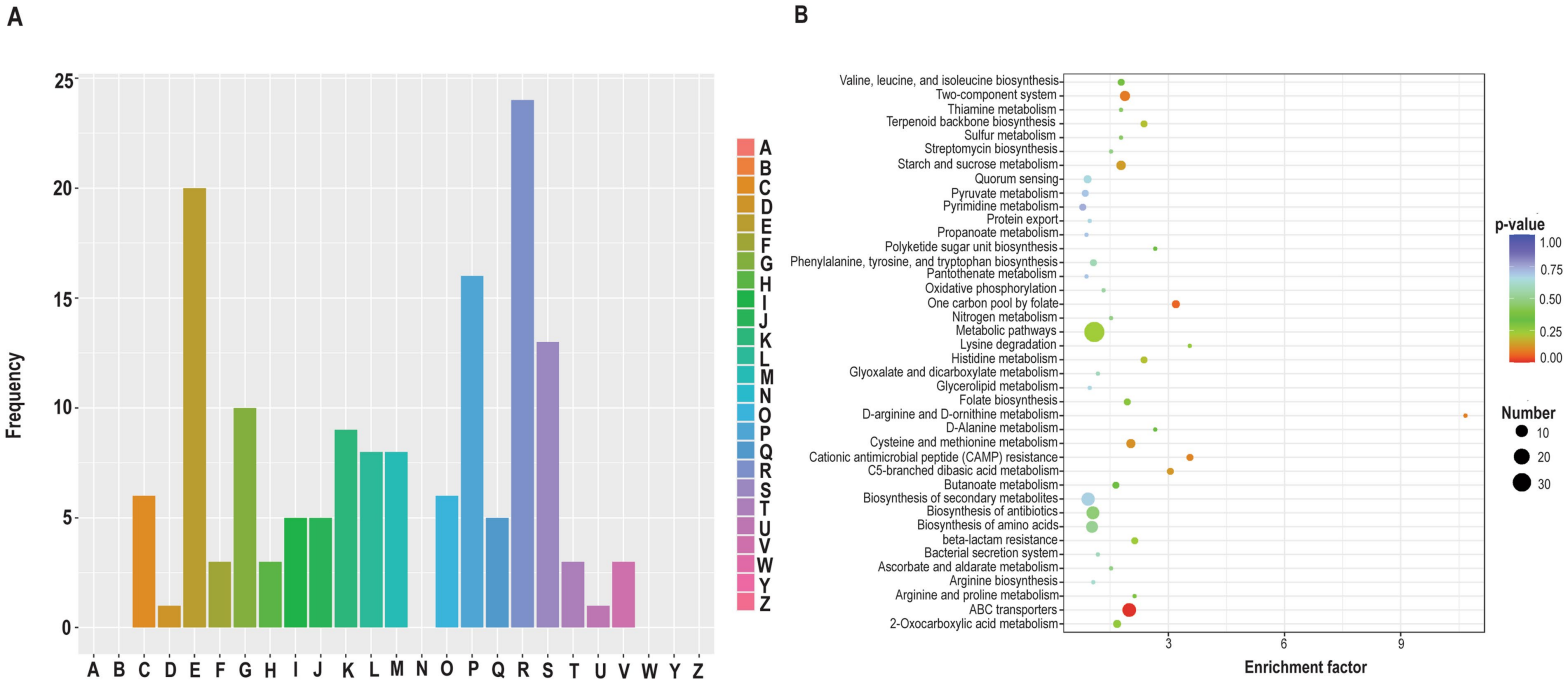
A comparative analysis of the DEPs between *S. mutans* wild-type and *ΔhlyX* cultures. Proteins were considered significantly differentially expressed if they met the criteria: |log_2_(fold change)| >log_2_(1.2) and *p* < 0.05, with *p*-values calculated using a two-tailed *t*-test method. **(A)** Classification of DEPs annotated in the cluster of orthologous groups (COGs) database (https://www.ncbi.nlm.nih.gov/COG/). The *x*-axis represents COG terms and *y*-axis represents protein count in each COG functional class (A–W, Y, Z). A: RNA processing and modification (0); B: Chromatin structure and dynamics (0); C: Energy production and conversion (6); D: Cell cycle control, cell division, chromosome partitioning (1); E: Amino acid transport and metabolism (20); F: Nucleotide transport and metabolism (3); G: Carbohydrate transport and metabolism (10); H: Coenzyme transport and metabolism (3); I: Lipid transport and metabolism (5); J: Translation, ribosome structure, and biogenesis (5); K: Transcription (9); L: Replication, recombination and repair (8); M: Cell wall/membrane/envelope biogenesis (8); N: Cell motility (0); O: Posttranslational modification, protein turnover, chaperones (6); P: Inorganic ion transport and metabolism (16); Q: Secondary metabolites biosynthesis, transport and catabolism (5); R: General function prediction only (24); S: Function unknown (13); T: Signal transduction mechanisms (3); U: Intracellular trafficking, secretion and vesicular transport (1); V: Defense mechanisms (3); W: Extracellular structure (0); Y: Nuclear structure (0); and Z: Cytoskeleton (0). The numbers in parentheses include the number of proteins. **(B)** KEGG enrichment analysis of the DEPs. The *x*-axis displays the enrichment factor, and the *y*-axis represents the pathway name. The number of proteins and the −log10(*p*-value) are indicated by the size and color of the dots, respectively. Protein pathway annotation was performed using the KEGG database. KOBAS 3.0 was employed to assign KEGG pathway descriptions to the differentially expressed proteins (DEPs). Enrichment analysis was conducted using Fisher’s exact test to evaluate the significance of DEP enrichment against the background of all identified proteins. Pathways with a corrected *p*-value <0.05 were considered statistically significant. Additionally, DEPs were classified into five major functional classes based on KEGG pathway definitions, excluding those used solely for enrichment analysis.

To explore the overlap between the phenotypic profiles of MgCl_2_-treated wild-type and Δ*hlyX* strains, we performed comparative proteomic analysis on Δ*hlyX* cells grown in THYE with or without 20 mM MgCl_2_ and compared the results to wild-type profiles. Approximately 1,500 proteins were identified, with 162 differentially expressed in Δ*hlyX* (105 upregulated, 57 downregulated; Supplementary Table S3), mirroring the upregulation trend seen in Mg^2+^-treated wild-type cells. GO up and down (Supplementary Figure S8), and enrichment analyses indicated that DEPs were enriched in transport, metal-ion binding (biological process and molecular function) (Supplementary Figures S10A,B), alongside those localized in the membrane (cellular component) (Supplementary Figure S10C), similar to the MgCl_2_-treated wild-type. COG analysis showed enrichment in amino acid, ion, and carbohydrate metabolism, with fewer signal transduction proteins affected in Δ*hlyX* (Figure 6A). KEGG pathway analysis highlighted ABC transporters and two-component systems among the top 40 enriched pathways (Figure 6B).

To investigate the molecular basis of the phenotypic similarity between MgCl_2_-treated wild-type cells and the Δ*hlyX* mutant, we compared their differentially expressed proteins (DEPs) relative to untreated wild-type cells. Thirty-five DEPs were shared between the two conditions, with 28 showing consistent up- or downregulation (Supplementary Table S3), supporting the observed phenotypic resemblance. Shared upregulated DEPs included DltC, several rhamnose-glucose polysaccharide (Rgp) biosynthetic proteins, an acyl carrier protein (Smu_1743), the polyamine transporter PotB, a putative histidine kinase (Smu_44), and a biofilm/competence-associated protein (Smu_63c). Downregulated DEPs included amino acid transporters encoded by Smu_932, Smu_933, Smu_935, and Smu_936. These overlaps suggest that both Mg^2+^ supplementation and HlyX deletion activate similar regulatory pathways, particularly those related to membrane processes, transport, and stress adaptation.

A direct comparison of MgCl₂-treated wild-type and untreated Δ*hlyX* proteomes revealed 229 DEPs (Supplementary Table S4). The Δ*hlyX* strain showed higher expression of several metal transporters (e.g., Smu_2057c, SloA, SloC, AdcA, PotA, PotC, TrkH) and two-component system proteins (e.g., Smu_44, Smu_46, Smu_660, LytS, CiaH). However, cell wall-anchored proteins (WapA, WapE, SpaP) were upregulated only in MgCl_2_-treated wild-type cells, indicating that Mg^2+^ uniquely promotes cell wall remodeling. Interestingly, despite overall elevated metal transporter expression in Δ*hlyX*, Smu_2057c was less abundant than in MgCl_2_-treated wild-type cells, potentially explaining the Δ*hlyX* strain’s increased Zn^2+^ sensitivity.

**Figure 7:**
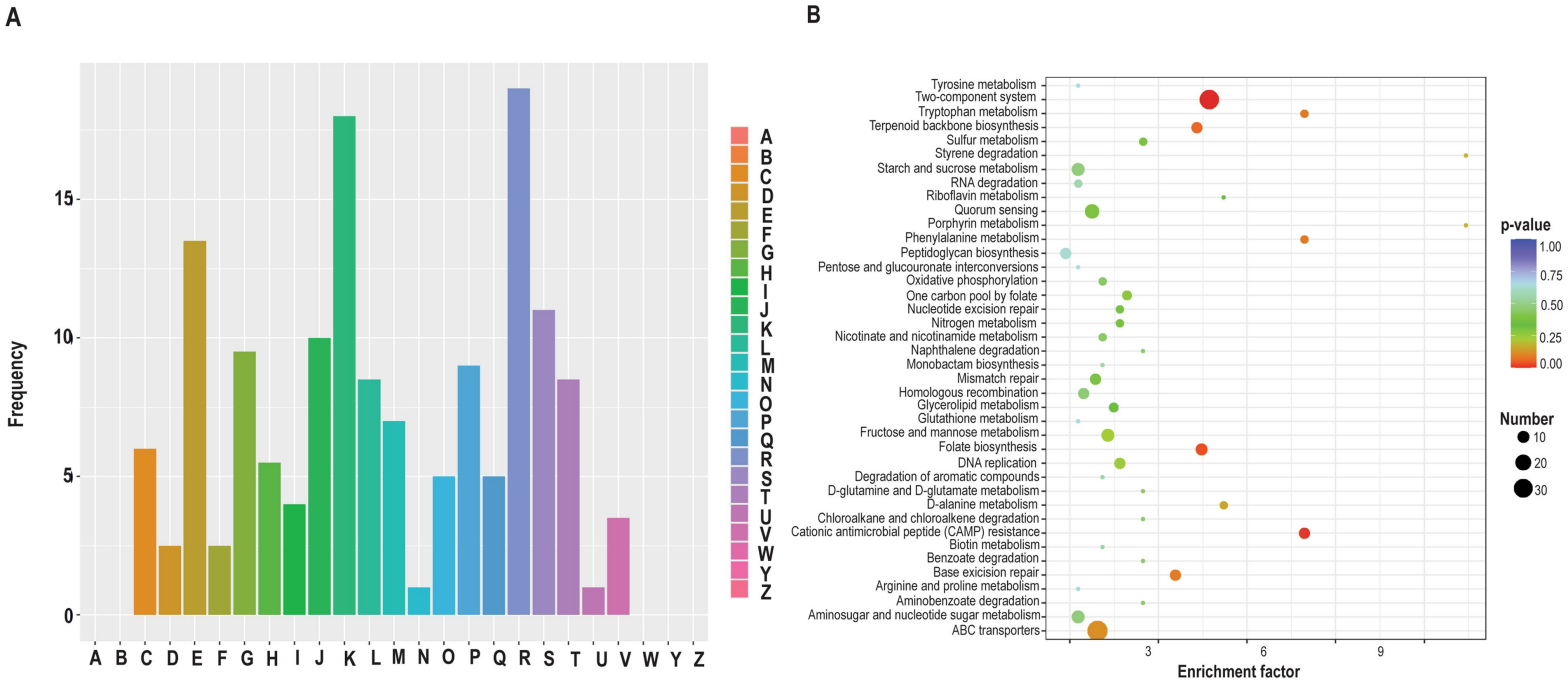
A comparative analysis of DEPs between MgCl_2_-treated *S. mutans* wild-type and *ΔhlyX* cultures. Proteins were considered significantly differentially expressed if they met the criteria: |log_2_(fold change)| >log_2_(1.2) and *p* < 0.05, with *p*-values calculated using a two-tailed *t*-test method. **(A)** Classification of DEPs annotated in cluster of orthologous groups (COGs) database (https://www.ncbi.nlm.nih.gov/COG/). The *x*-axis represents COG terms and *y*-axis represents protein count in each COG functional class (A–W, Y, Z). A: RNA processing and modification (0); B: Chromatin structure and dynamics (0); C: Energy production and conversion (12); D: Cell cycle control, cell division, chromosome partitioning (5); E: Amino acid transport and metabolism (27); F: Nucleotide transport and metabolism (5); G: Carbohydrate transport and metabolism (19); H: Coenzyme transport and metabolism (11); I: Lipid transport and metabolism (8); J: Translation, ribosome structure, and biogenesis (20); K: Transcription (36); L: Replication, recombination and repair (17); M: Cell wall/membrane/envelope biogenesis (14); N: Cell motility (2); O: Posttranslational modification, protein turnover, chaperones (10); P: Inorganic ion transport and metabolism (18); Q: Secondary metabolites biosynthesis, transport and catabolism (10); R: General function prediction only (38); S: Function unknown (22); T: Signal transduction mechanisms (17); U: Intracellular trafficking, secretion and vesicular transport (2); V: Defense mechanisms (7); W: Extracellular structure (0); Y: Nuclear structure (0); and Z: Cytoskeleton (0). The numbers in parentheses include the number of proteins. **(B)** KEGG enrichment analysis of the DEPs. The *x*-axis displays the enrichment factor, and the *y*-axis represents the pathway name. The number of proteins and the −log10(*p*-value) are indicated by the size and color of the dots, respectively. Protein pathway annotation was performed using the KEGG database. KOBAS 3.0 was employed to assign KEGG pathway descriptions to the differentially expressed proteins (DEPs). Enrichment analysis was conducted using Fisher’s exact test to evaluate the significance of DEP enrichment against the background of all identified proteins. Pathways with a corrected *p*-value <0.05 were considered statistically significant. Additionally, DEPs were classified into five major functional classes based on KEGG pathway definitions, excluding those used solely for enrichment analysis.

To explore Mg^2+^-excess toxicity, we compared the proteomes of MgCl_2_-treated wild-type and Δ*hlyX* strains, identifying 318 DEPs (Supplementary Table S5), with balanced up- and downregulation. GO analysis revealed enrichment in nutrient transport, metabolism, nucleotide binding, and membrane-localized proteins (Supplementary Figures S8, S11A–C). COG classification highlighted transcription, amino acid and carbohydrate metabolism as major affected categories (Figure 7A). Notably, translation, ion transport, and signal transduction were more impacted in MgCl_2_-treated strains than in untreated controls (Figures 6A, 7A). KEGG analysis further emphasized two-component systems and cationic antimicrobial peptide (CAMP) resistance pathways (Figure 7B). Overall, Mg^2+^ supplementation in the absence of HlyX induces a compensatory upregulation of membrane-associated proteins involved in envelope biogenesis, transport, and signaling, enhancing stress tolerance but also revealing vulnerabilities in the Δ*hlyX* background.

### Mg^2+^-dependent stress tolerance involves the modulation of the *S. mutans* lipidome

Proteomic analysis of MgCl_2_-treated wild-type *S. mutans* and both untreated and MgCl_2_-treated Δ*hlyX* cultures showed consistent upregulation of membrane-localized proteins involved in transport, signal transduction, and cell envelope biogenesis, relative to untreated wild-type cells. Notably, proteins associated with membrane biogenesis, such as YajC, SecE, YidC2, and YlxM, were abundant in both MgCl_2_-treated wild-type and Δ*hlyX* strains. In addition to the membrane proteins, *S. mutans* and other bacteria have been shown to modulate their lipid composition, which is relevant for the localization, and structure–function of membrane proteins (Mishra et al., 2023; Dowhan et al., 2019; MacGilvray et al., 2012).

**Figure 8:**
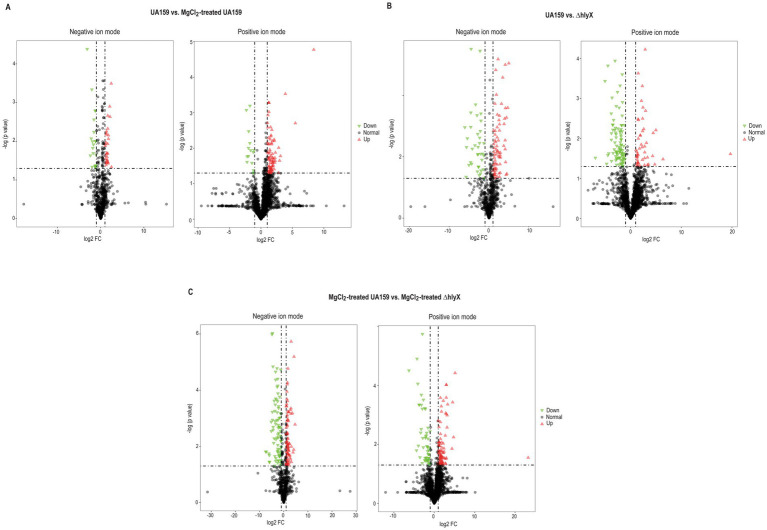
Volcano plots showing the distribution of various lipid species (lipids with VIP >1.5, FC >2 and *p*-value <0.05 were deemed significant). **(A)** Untreated wild-type versus MgCl_2_-treated wild-type, **(B)** wild-type versus *ΔhlyX*, and **(C)** MgCl_2_-treated wild-type versus MgCl_2_-treated *ΔhlyX*. Raw LC-MS/MS lipid data were acquired and aligned using LipidSearch software (Thermo) based on *m*/*z* values and retention times of ion signals. Data from both ESI-negative and ESI-positive modes were merged and imported into SIMCA-P (version 14.1) for multivariate analysis. Principal component analysis (PCA) was first applied as an unsupervised method for data visualization and outlier detection. Subsequently, supervised regression modeling was performed using partial least squares discriminant analysis (PLS-DA) and orthogonal partial least squares discriminant analysis (OPLS-DA) to identify potential biomarkers. Candidate biomarkers were filtered and confirmed by combining variable importance in projection (VIP) scores (VIP >1.5), fold-change (FC >2), and Student’s *t*-test (*p* < 0.05). Red triangles represent the upregulated lipids, while green triangles represent the downregulated species.

To investigate whether Mg^2+^ supplementation or HlyX deletion modulates the lipidome in *S. mutans*, comprehensive lipidomic analyses were performed on wild-type and Δ*hlyX* strains, with and without MgCl_2_ supplementation. Using UPLC-MS in both positive and negative ion modes, over 2,000 and 700 lipid species were identified, respectively (Supplementary Tables S6, S7). Three pairwise comparisons were conducted: (1) untreated vs. MgCl_2_-treated wild-type, (2) wild-type vs. Δ*hlyX*, and (3) MgCl_2_-treated wild-type vs. MgCl_2_-treated Δ*hlyX*. Unsupervised principal component analysis (PCA) revealed distinct lipidomic profiles across all comparisons, except between untreated and MgCl₂-treated wild-type samples in positive ion mode (Supplementary Figures S12A,B). These findings were validated using partial least squares-discriminant analysis (PLS-DA), which confirmed clear group separation (Supplementary Figures S12C,D). Differential lipids were identified based on VIP >1.5, FC >2, and *p* < 0.05 (Supplementary Tables S8–S13 and Figures 8A–C).

Volcano plots (Figure 8A and Supplementary Tables S8, S9) revealed a general increase in lipid abundance in MgCl_2_-treated wild-type *S. mutans*, suggesting that moderate Mg^2+^ supplementation promotes lipid biosynthesis, consistent with its role in supporting growth and membrane development. In contrast, Δ*hlyX* strains, independent of MgCl_2_ treatment, did not show a similar global increase in various lipid species (Figures 8B,C). Instead, many lipid species, particularly neutral lipids in positive ion mode, were reduced, indicating exposure to environmental stress.

**Figure 9:**
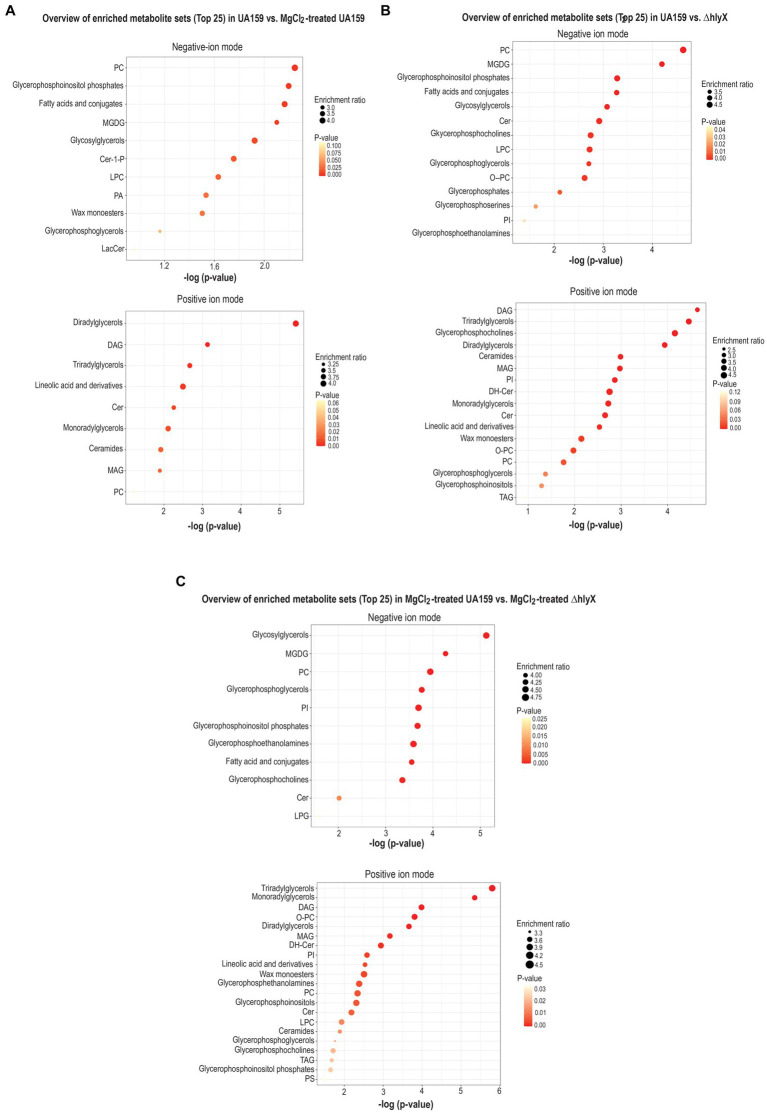
Pathway enrichment analysis of lipids **(A)** wild-type (untreated vs. MgCl_2_-treated), **(B)** wild-type vs. *ΔhlyX*, and **(C)** MgCl_2_-treated wild-type vs. MgCl_2_-treated *ΔhlyX*. A correlation network diagram based on the KEGG databases and MetaboAnalyst was made by importing all significant lipids to obtain the categorical annotations, including pathways. The size of the dots represents the enrichment score, and the color intensity is representative of the *p*-value.

Across all samples, glycolipids and glycerophospholipids dominated in negative ion mode, while glycerolipids were the most abundant lipids in the positive ion mode (Supplementary Tables S8–S13). Several lipid species exhibited differential abundance between untreated and MgCl₂-treated wild-type *S. mutans* cultures, specifically monogalactosyldiacylglycerol (MGDG) and phosphatidylglycerol (PG). Among glycerolipids, diradylglycerols (DG) were most abundant, followed by triradylglycerols (TG) and monoradylglycerols (MG). The relative proportion of most glycerolipids was higher in MgCl_2_-treated cells, indicating enhanced glycerolipid biosynthesis under Mg^2+^-supplemented conditions (Supplementary Tables S9, S11, S13). MgCl_2_ treatment also led to a shift in fatty acid saturation profiles. Specifically, there was an increase in saturated fatty acid side chains in PG, accompanied by a decrease in monounsaturated fatty acids. In contrast, MGDG species with polyunsaturated fatty acids were elevated in MgCl_2_-treated wild-type cells (Supplementary Table S9). Mapping significantly altered lipids to KEGG biosynthetic pathways revealed more than a three-fold enrichment in several lipid classes, including glycerolipids (DG, DAG, TG, MG, MAG), glycerophospholipids (e.g., phosphatidylcholine, glycerophosphoinositol phosphates), and glycolipids (e.g., MGDG, glycosylglycerols) (Figure 9A). The lipidomic profile of wild-type cells resembled that of a log-phase culture, as reported previously in the literature (Custer et al., 2014).

Significant reductions in the levels of anionic phospholipids, phosphatidylglycerol (PG) and phosphatidylinositol (PI), were observed in the Δ*hlyX* strain (Supplementary Tables S11, S13). Notably, a greater number of lipid species containing polyunsaturated fatty acid chains were identified among the significantly altered lipids in this mutant. Interestingly, MgCl_2_-treated Δ*hlyX* cultures exhibited a multiple-fold increase in PI levels compared to MgCl_2_-treated wild-type cells (Supplementary Tables S11, S13), while PG and phosphatidylcholine (PC) species with saturated fatty acid side chains were reduced. These findings suggest that Mg^2+^ supplementation in the absence of HlyX alters the balance of anionic phospholipids, potentially affecting membrane charge and fluidity. Notably, deletion of *hlyX* led to a marked enrichment of triglycerides (TG) under both untreated and MgCl_2_-treated conditions when significant lipids were mapped to KEGG pathways (Figures 9B,C). As TGs are commonly associated with lipid storage, their accumulation in Δ*hlyX* cells suggests a physiological shift toward a stress-adapted state rather than a growth-promoting condition. These findings highlight the impact of Mg^2+^ availability and HlyX function on lipid class distribution and fatty acid composition, reinforcing the role of membrane remodeling in bacterial stress adaptation. Lipidomic samples were collected during logarithmic-phase growth, which may explain the absence of cardiolipin—previously reported as the most abundant phospholipid in *S. mutans* membranes under stationary-phase conditions (Morales-Aparicio et al., 2020).

To further understand the metabolic implications of Mg^2+^ perturbation, significantly impacted lipids from each pairwise comparison was mapped to KEGG pathways. A consistent theme across comparisons was the predicted downregulation of the enzyme encoded by *smu_775c*, which is involved in the lipoteichoic acid (LTA) biosynthetic pathway (Supplementary Figure S13). Homology analysis identified SMU_775c as a Mn^2+^-binding protein, suggesting that elevated intracellular Mg^2+^ may competitively inhibit Mn^2+^-dependent enzymes. Since LTA biosynthesis utilizes neutral glycerolipids, its downregulation likely contributes to the observed shifts in glycerolipid metabolism in MgCl_2_-treated cultures.

### Mg^2+^ modulates the susceptibility to antibiotics

Distinct hypotheses have been proposed to explain the role of Mg^2+^ in modulating antibiotic efficacy in both actively growing and dormant bacterial cells. Dormant cells, or persisters, typically exhibit high tolerance to antibiotics, as these agents target biosynthetic processes—such as peptidoglycan and protein synthesis—that are inactive in non-replicating cells. Recent studies in *B. subtilis* have shown that Mg^2+^ import mitigates membrane hyperpolarization, contributing to antibiotic tolerance in actively growing cells (Lee et al., 2019). Building on our findings regarding the roles of Mg^2+^ and HlyX in *S. mutans* stress adaptation, we hypothesized that these factors may influence antibiotic susceptibility by supporting bacterial growth. To test this, we assessed the minimum inhibitory concentrations (MICs) of various antibiotics using Etest strips on THYE agar, with and without 20 mM MgCl_2_ supplementation. In wild-type cells, MgCl_2_ treatment led to a notable reduction in MICs for several cell wall-targeting antibiotics (Table 3), suggesting enhanced antibiotic efficacy under Mg^2+^-rich conditions. In contrast, the Δ*hlyX* mutant exhibited a variable response: MICs for bacitracin, daptomycin, and vancomycin increased, while those for amoxicillin and clindamycin decreased relative to the wild-type. When grown in MgCl_2_-supplemented medium, Δ*hlyX* cells showed reduced MICs for most antibiotics, except clindamycin, compared to unsupplemented conditions. To further explore the role of Mg^2+^, we included the Δ*rpmH* strain in our analysis. This mutant, characterized by low intracellular Mg^2+^ and elevated levels of other metal ions (Na^+^, K^+^, Fe^2+^, Cu^2+^), exhibited no significant reduction in MICs except for amoxicillin and vancomycin. Interestingly, MgCl_2_ supplementation restored growth in Δ*rpmH* but also increased MICs, indicating enhanced antibiotic tolerance. Despite achieving wild-type Mg^2+^ levels, the strain retained elevated concentrations of other metals (except Mn^2+^), which may contribute to its altered antibiotic response. Overall, these findings support the hypothesis that Mg^2+^ availability influences antibiotic efficacy, particularly in rapidly growing cells, and that metal ion homeostasis broadly shapes bacterial susceptibility to antimicrobial agents.

**Table 3:** Antibiotic MICs determined using Etest strips against *S. mutans* strains.

Antibiotics	Target	MIC as determined by Etest strips (mg mL^−1^)					
		UA159		*ΔhlyX*		*ΔrpmH*	
		0 mM MgCl_2_	20 mM MgCl_2_	0 mM MgCl_2_	20 mM MgCl_2_	0 mM MgCl_2_	20 mM MgCl_2_
Amoxicillin	Peptidoglycan (cell wall)	0.125	0.064	0.094	0.047	0.016	0.023
Bacitracin	Cell wall	16	6	32	24	16	24
Clindamycin	Protein synthesis	0.094	0.094	0.047	0.064	FG*	FG*
Daptomycin	Cell membrane	4	4	12	3	4	3
Vancomycin	Cell wall	1	0.75	3	1.5	0.5	1

The data shown is the mean of three independent experiments. *FG, fully grown on the plate like a lawn.

## Discussion

Changes in salivary Mg^2+^ levels expose the oral bacteriome, particularly the genus *Streptococcus*, to significant fluctuations in Mg^2+^. Despite the abundance and variability of Mg^2+^ in the oral cavity, its homeostasis has not garnered as much attention as other divalent metals like Fe^2+^, Mn^2+^, Cu^2+^, and Zn^2+^ (reviewed in Richards et al., 2017). These divalent metal ions are crucial for the growth and metabolism of bacteria, similar to Mg^2+^; however, they are required in smaller quantities than Mg^2+^, and minor fluctuations in the intracellular concentrations of these metals can often lead to toxicity. The identification of a putative Mg^2+^ efflux pump in various bacterial species, including *S. typhimurium* (Gibson et al., 1991), *B. subtilis* (Cheng et al., 2018), and *S. aureus* (Gaballa et al., 2003), suggests that although Mg^2+^ is a macronutrient, elevated levels above a certain threshold can be toxic to bacteria, like other divalent cations. This study demonstrates that oral streptococci exhibit a lower tolerance to exogenous MgCl_2_ than pyogenic streptococci, which are adapted to higher serum Mg^2+^ levels. Gram-positive bacteria, including *B. subtilis* and *S. aureus*, also tolerate ≥100 mM Mg^2+^ salts in growth media. Notably, *S. mutans* maintained intracellular Mg^2+^ concentrations four to five times lower than *B. subtilis*, suggesting a reduced Mg^2+^ requirement for growth and survival. These findings underscore the significance of Mg^2+^ regulation in oral streptococci, suggesting that their sensitivity to Mg^2+^ may reflect adaptation to the relatively low and variable Mg^2+^ concentrations present in the oral environment.

In *B. subtilis*, elevated intracellular Mg^2+^ levels are supported by at least three characterized transporters—MgtE, CorA, and CitM—that likely function as Mg^2+^ importers (Ajdić et al., 2002). A homolog of the MgtA/B transporter family has also been identified in *B. subtilis*, although its role in Mg^2+^ uptake remains unclear (Ajdić et al., 2002). In contrast, among the *Streptococcus* species examined in this study, only *S. sanguinis* SK36 possesses a homolog of *mgtE*. *S. mutans* and closely related species (*S. ratti*, *S. downei*, *S. criceti*, and *S. sobrinus*) are unique in harboring a single *corA* homolog. Those belonging to the sanguinis, mitis, salivarius, anginosus, and pyogenic groups of streptococci contain two distinct paralogs of CorA. Functional studies using transposon mutagenesis and CRISPR-Cas9 have demonstrated that *corA* is essential in *S. mutans* (Shields et al., 2018; Shields et al., 2020). Homology searches also identified MgtA/B—like transporters in representative *Streptococcus* genome. However, the essentiality of *corA* in *S. mutans* suggests that it may serve as the sole Mg^2+^ importer in this species. This limited transporter repertoire likely contributes to the lower intracellular Mg^2+^ levels observed in *S. mutans* compared to other bacteria, such as *B. subtilis*, which possess multiple Mg^2+^ uptake systems.

Although *S. mutans* and related streptococci are distinct from other *Streptococcus* spp. in possessing only a single Mg^2+^ uptake protein, they all encode a homolog of the *B. subtilis* magnesium efflux protein MpfA, annotated as hemolysin (HlyX). Deletion of *hlyX* in *S. mutans* and *S. sanguinis* resulted in phenotypes similar to the *B. subtilis* Δ*mpfA* mutant, including increased sensitivity to Mg^2+^, enhanced resistance to osmotic stress, and improved tolerance to divalent metal toxicity (notably Co^2+^ and Zn^2+^). However, unlike *B. subtilis*, the *S. mutans* Δ*hlyX* strain did not exhibit elevated intracellular Mg^2+^ levels. Supplementation with MgCl_2_ restored Mg^2+^ concentrations in Δ*hlyX* cells to wild-type levels but did not exceed them (Table 2). One potential explanation for the reduction in intracellular Mg^2+^ concentrations in the untreated Δ*hlyX* strain may be the existence of alternative, yet unidentified, mechanisms of Mg^2+^ efflux induced by the removal of *hlyX*. ICP-MS analysis and growth assays in THYE ± 20 mM MgCl_2_ indicate that *S. mutans* maintains an upper intracellular Mg^2+^ threshold of approximately 0.5–0.6 μg/mL/mg protein. A 50% reduction in this level did not disrupt homeostasis, further supporting the hypothesis that *S. mutans* has a relatively low Mg^2+^ requirement. The essentiality of the *corA* gene and the stable growth of the Δ*hlyX* strain under reduced Mg^2+^ conditions further support the notion that *S. mutans* is adapted to thrive in environments with limited magnesium availability.

Surprisingly, the intracellular Mg^2+^ concentration in the untreated Δ*rpmH* strain was comparable to that of the Δ*hlyX* mutant. However, the Δ*rpmH* strain exhibited a distinct growth phenotype. The *rpmH* gene encodes ribosomal protein L34, and in *B. subtilis*, Mg^2+^ supplementation has been shown to partially restore the growth defect of the Δ*rpmH* mutant. Similarly, in *S. mutans*, the growth defect and intracellular Mg^2+^ concentration of the Δ*rpmH* strain were restored upon supplementation with 20 mM MgCl_2_. Notably, ICP-MS analysis revealed that the Δ*rpmH* mutant accumulated significantly higher levels of other intracellular cations, approximately 3-fold to 5-fold greater than those in the Δ*hlyX* and wild-type strains under identical conditions. This elevated cation burden became functionally relevant when the Δ*rpmH* strain was tested for sensitivity to osmotic and divalent metal stress. The mutant showed heightened sensitivity to KCl-induced osmotic stress and Co^2+^ and Zn^2+^ toxicity, likely due to its elevated intracellular metal concentrations. Supplementation with MgCl_2_ not only restored growth but also conferred protection against these stressors (Figure 4). Taken together, the growth profile and intracellular Mg^2+^ concentration of both wild-type and mutant strains suggest that the physiological effects of Mg^2+^ on bacterial growth and stress tolerance cannot be considered in isolation. Instead, they must be evaluated in the context of the broader ionic environment, particularly in relation to biologically relevant cations such as K^+^, Na^+^, Fe^2+^, Zn^2+^, Cu^2+^, and Co^2+^.

Protein metalation generally follows the Irving–Williams series (Mg^2+^ < Mn^2+^ < Fe^2+^ < Co^2+^ < Ni^2+^ < Cu^2+^ > Zn^2+^), placing Mg^2+^ at the lowest binding affinity (Irving and Williams, 1953). However, metalation is also influenced by intracellular metal availability, and weak binders like Mg^2+^ are typically not displaced by stronger competitors such as Zn^2+^ (Foster et al., 2022). The distinct growth phenotype of the Δ*rpmH* mutant, compared to Δ*hlyX* and MgCl₂-treated Δ*rpmH*, can be explained by the relative bioavailability of other metals in relation to Mg^2+^. Metal bioavailability is regulated by the coordinated activity of importers, efflux pumps, metallochaperones, and transcriptional regulators. Proteomic analysis of wild-type and Δ*hlyX* strains revealed differential expression of several metal transporters and regulators. Notably, the CorA homolog (*Smu_1852*) was upregulated (~1-fold increase) in MgCl_2_-treated Δ*hlyX* cells compared to treated wild-type cells, suggesting enhanced Mg^2+^ import to maintain homeostasis in the absence of HlyX. This may reflect the induction of an alternative Mg^2+^ efflux mechanism. Additionally, CovR (Smu_1924), a known repressor of Mg^2+^-responsive genes in *S. pyogenes* (Gryllos et al., 2007), as downregulated (0.38-fold) in Δ*hlyX*, indicating reduced repression of Mg^2+^ import. The Δ*hlyX* strain also showed downregulation of the Mn^2+^ uptake repressor SloR and the Zn^2+^ efflux pump (Smu_2057c), suggesting increased intracellular Mn^2+^ and Zn^2+^ under Mg^2+^-limited conditions. In MgCl_2_-treated Δ*hlyX* cells, levels of Mn^2+^ importers (SloA, SloC) and Zn^2+^ efflux pump was reduced, mirroring the high Zn^2+^ and unchanged Mn^2+^ levels observed in Δ*rpmH*. These findings highlight the complex interplay between Mg^2+^ and other biologically relevant cations (e.g., K^+^, Na^+^, Fe^2+^, Zn^2+^, Cu^2+^, Co^2+^) in shaping bacterial growth and stress responses.

Lipidomic analysis further supports the protective role of Mg^2+^ against environmental stress in *S. mutans*. Previous studies have shown that exposure to acidic conditions (pH 5) leads to an increase in monounsaturated fatty acids in *S. mutans* (Fozo and Quivey, 2004). Consistent with this, our data revealed elevated levels of unsaturated fatty acids, glycerophospholipids, and glycolipids in cells treated with MgCl_2_. Glycolipids, classified as polar lipids, are typically synthesized under phosphate-limiting conditions (Peng et al., 2019; Peng and Miao, 2020). Similar lipidomic shifts, including increased monogalactosyldiacylglycerol (MGDG) levels, have been reported in *Synechococcus* under phosphate starvation (Peng and Miao, 2020). The lipid profiles of MgCl₂-treated wild-type and Δ*hlyX* strains resembled those observed in phosphate-starved bacteria, suggesting a potential link between Mg^2+^ availability and phosphate metabolism. Proteomic analysis of Δ*hlyX* cells revealed a decrease in the abundance of several two-component system histidine kinases, which may reflect phosphate limitation. One plausible explanation is that elevated Mg^2+^ levels neutralize intracellular phosphate, reducing its bioavailability. In response, the cytoplasmic membrane may compensate by increasing the synthesis of polar lipids such as glycolipids and phosphoglycerolipids. These findings highlight a previously underappreciated connection between Mg^2+^ homeostasis and lipid remodeling, suggesting that Mg^2+^ not only contributes to stress tolerance but also influences membrane composition in response to nutrient availability.

Interestingly, Mg^2+^ supplementation did not confer protection to *S. mutans* against antibiotic stress, particularly from cell wall-targeting antibiotics. Treatment with MgCl_2_ led to increased abundance of several cell wall-anchored proteins (SpaP, WapA, WapE, and GbpC), rhamnose-glucose polysaccharide (Rgp) biosynthesis proteins, and Pbp2a, which is involved in cell surface modification. This upregulation is associated with enhanced growth in wild-type cells under Mg^2+^-rich conditions. However, actively growing cells are more susceptible to antibiotics, which often target biosynthetic pathways. Penicillin-binding proteins (Pbps) are the primary targets of β-lactam antibiotics in bacteria. Among these, Pbp1a, whose function remains poorly defined but is known to have reduced β-lactam affinity in *S. pneumoniae*, was the only Pbp upregulated in Mg^2+^-treated wild-type cells (Zhao et al., 2000). In contrast, deletion of *hlyX* resulted in increased minimum inhibitory concentrations (MICs) for bacitracin, daptomycin, and vancomycin (Table 3), indicating enhanced antibiotic tolerance. This suggests that HlyX plays a role in modulating antibiotic susceptibility, potentially through its influence on Mg^2+^ homeostasis. Notably, Δ*hlyX* cultures grown in MgCl₂-supplemented medium exhibited increased sensitivity to various antibiotics, despite the elevated Mg^2+^ levels. These findings imply that while Mg^2+^ promotes growth and cell wall biosynthesis, it may also increase vulnerability to antibiotics. Conversely, the absence of HlyX, coupled with low intracellular Mg^2+^, appears to induce a stress-adapted state that enhances antibiotic tolerance.

The findings of this study demonstrate that Mg^2+^ significantly impacts the growth, stress response, and antibiotic sensitivity of oral streptococci. These results suggest that Mg^2+^ may play a broader role in shaping the oral microbiome and maintaining oral health. Therefore, the potential prebiotic application of Mg^2+^ warrants further investigation, particularly in the context of dietary supplementation and its effects on oral microbial ecology. Future studies should focus on characterizing Mg^2+^ transporters and elucidating the mechanisms of Mg^2+^ homeostasis in oral bacteria to better understand how Mg^2+^ availability influences microbial physiology and host–microbe interactions.

## Data Availability

The data presented in the study are deposited in the ProteomeXchange partner MassIVE repository, accession number MSV000099768.

## Footnotes

1
http://www.ebi.ac.uk/GOA/
