# Supplementary material for: Magnesium modulates the stress responses of oral streptococci to environmental and antibiotic challenges by altering cell envelope and nutrient transport pathways
Source: Front Microbiol. 2026 Jan 6;16:1669039. doi: 10.3389/fmicb.2025.1669039 (PMC12815764; doi:10.3389/fmicb.2025.1669039)
Supplement: Supplementary file 2 [file Data_Sheet_1.docx]

**Supplemental data**

**Table S1: List of bacterial strains and plasmids used in this study.**

| **Strains** | **Genotype/description** | **Source/reference** |
| --- | --- | --- |
| *S. mutans* UA159 | Wild-type (serotype c) | [1] |
| *S. mutans* NG8 | Wild-type (serotype c) | Laboratory stock |
| *S. sobrinus* strain 6715 | Wild-type | Laboratory stock |
| *S. downei* MFe 28 | Wild-type | Laboratory stock |
| *S. mitis* SK132 | Wild-type | Laboratory stock |
| *S. oralis* SK92 | Wild-type | Laboratory stock |
| *S. gordonii* Dl-1 | Wild-type | Laboratory stock |
| *S. salivarius* K12 | Wild-type | Laboratory stock |
| *S. agalactiae* A909 | Wild-type | Laboratory stock |
| *S. pyogenes* NZ131 | Wild-type | Laboratory stock |
| SM2341 | UA159 *ΔhlyX::*aad9; Spc^R^ | This work |
| SM2824 | UA159 *ΔrpmH::ermB*; Erm^R^ | This work |
| SM3042 | UA159 *ΔhlyX::*aad9/*pIB166-hlyX-V5*; Spc^R^, Cam^R^ | This work |
| S. sanguinis SK36 | Wild type, Human plaque isolate | [2] |
| *Δssa1761* | SK36, insert-deletion of *ssa_1761* gene, Km^R^ | [3] |
| ***Escherichia coli*** | | |
| C2987 | *fhuA2 Δ(argF-lacZ)U169 phoA glnV44 f80Δ(lacZ)M15 gyrA96 recA1 relA1 endA1 thi-1 hsdR17* | NEB |
| **Plasmids** | | |
| pIB166 | *E. coli* - streptococci shuttle plasmid for gene expression in streptococci with P23 promoter, Cam^R^ | [4] |
| pIB166-hlyX-V5 | pIB166 containing V5-tagged hlyX subcloned between BamHI and HindIII, Cam^R^ | This work |


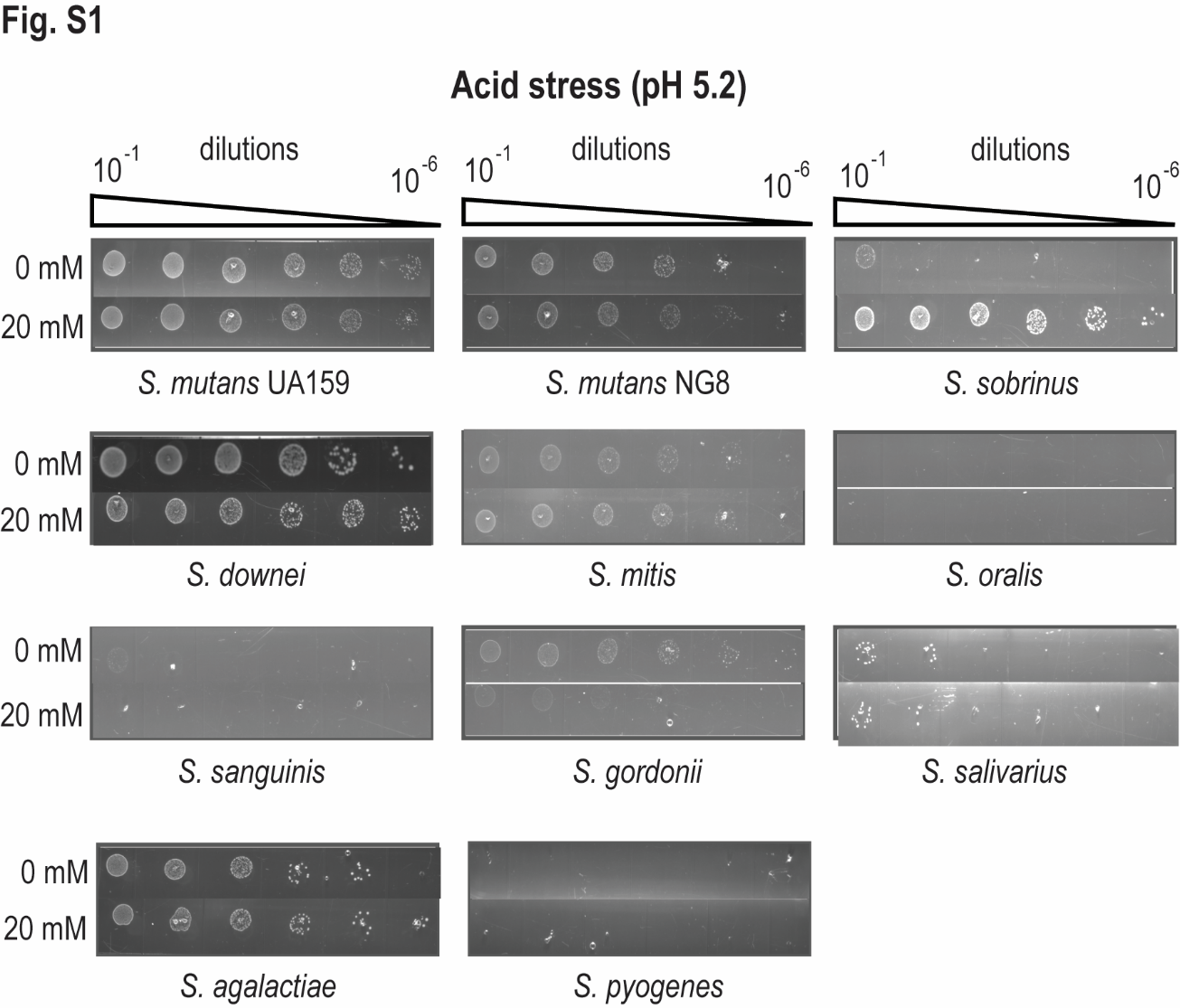


**Fig. S1: Effect of supplementation of the growth medium with MgCl₂ on the survival of various *Streptococcus* *spp.* under acid stress conditions.** Overnight cultures were diluted in BHI to an O.D.₆₀₀ of 0.2, followed by 10-fold serial dilutions. Aliquots (4 μL) of each dilution were spotted onto BHI agar plates adjusted to pH 5.2, with or without 20 mM MgCl₂. The dilutions ranged from 10⁻¹ (left) to 10⁻⁶ (right). Plates were incubated at 37 °C in a 5% CO₂ atmosphere for 48 hours. Representative images from four independent experiments are shown.


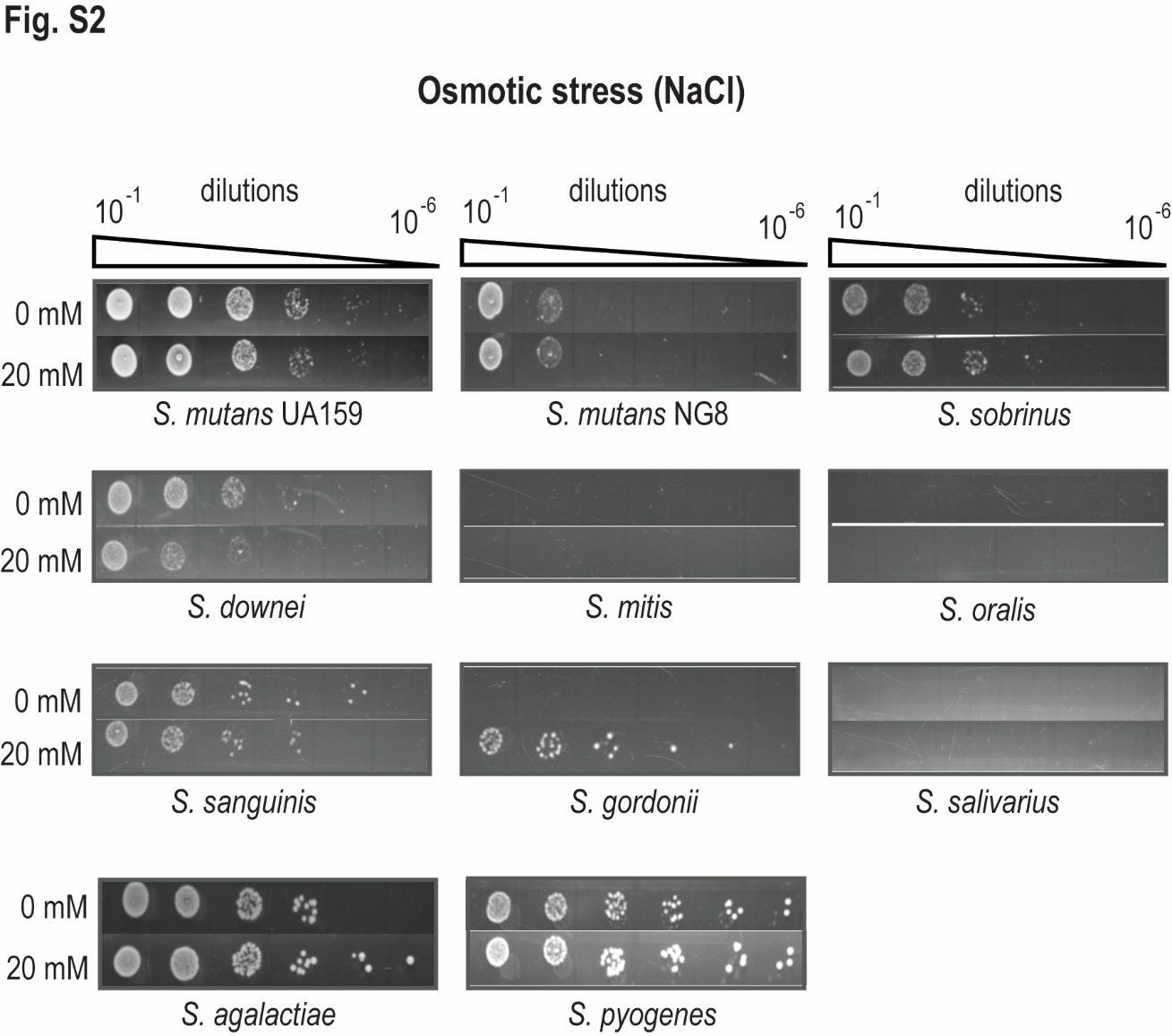


**Fig. S2: Effect of supplementation of the growth medium with MgCl₂ on the survival of various *Streptococcus* *spp.* when exposed to 0.5 M NaCl.** Overnight cultures of *Streptococcus spp.* were diluted in BHI to an O.D._600_ of 0.2, followed by 10-fold serial dilutions in BHI. Drops (4 μL) were spotted onto BHI plates containing 0.5 M NaCl ± 20 mM MgCl_2_, ranging from the 10^-1^ dilution (left) to the 10^-6^ dilution (right). Plates were incubated at 37 °C in a 5% CO_2_ atmosphere for 2 days before documentation. The images shown are representative of three independent experiments.


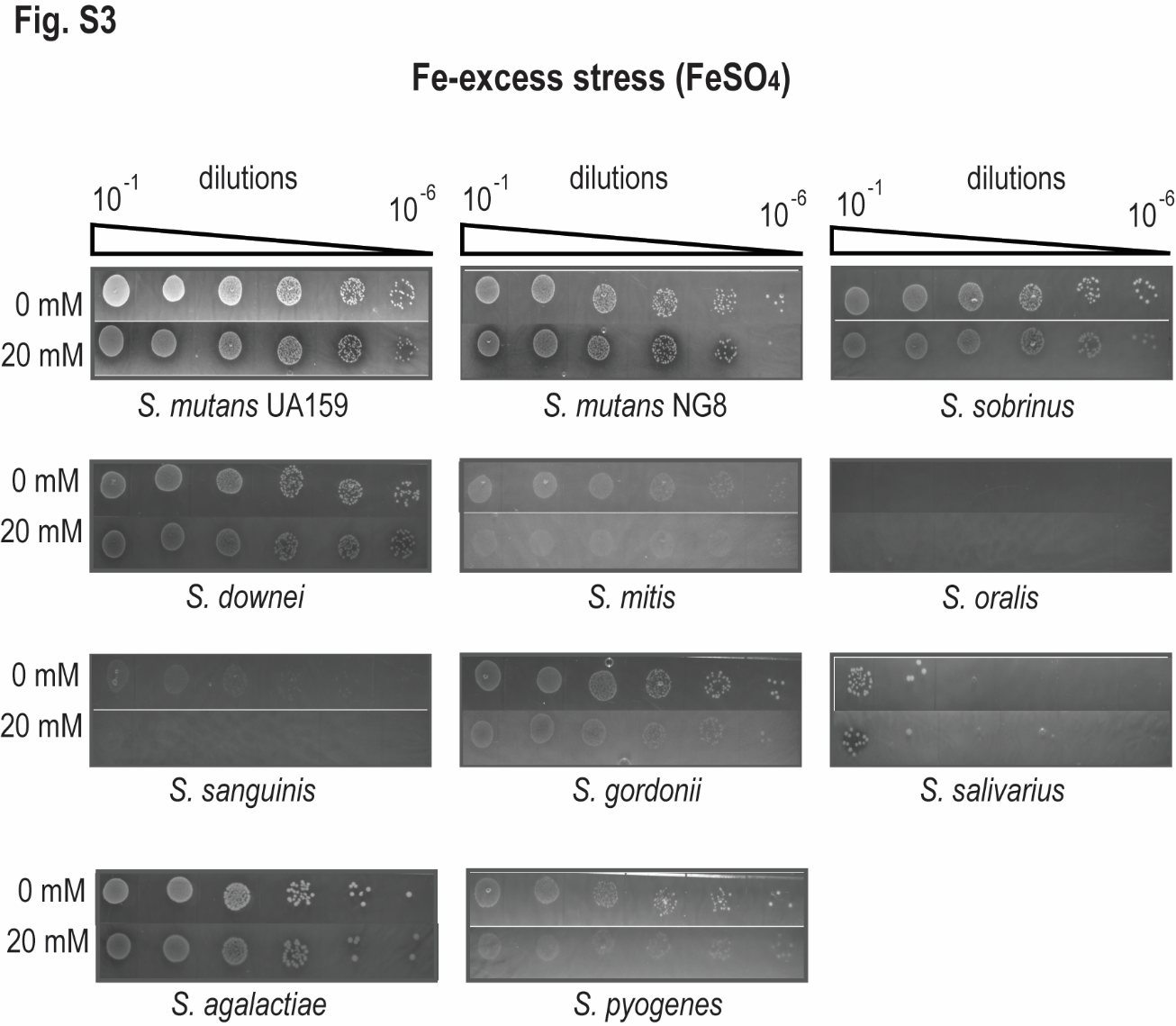


**Fig. S3: Impact of MgCl_2_ supplementation on the survival of *Streptococcus spp.* when exposed to 5 mM FeSO_4_.** Overnight cultures of *Streptococcus spp.* were diluted in BHI to an O.D._600_ of 0.2, followed by 10-fold serial dilutions in BHI. Drops (4 μL) were spotted onto BHI plates containing 5 mM FeSO_4_ ± 20 mM MgCl_2_, ranging from the 10^-1^ dilution (left) to the 10^-6^ dilution (right). Plates were incubated at 37 °C in a 5% CO_2_ atmosphere for 2 days before documentation. The images shown are representative of three independent experiments.


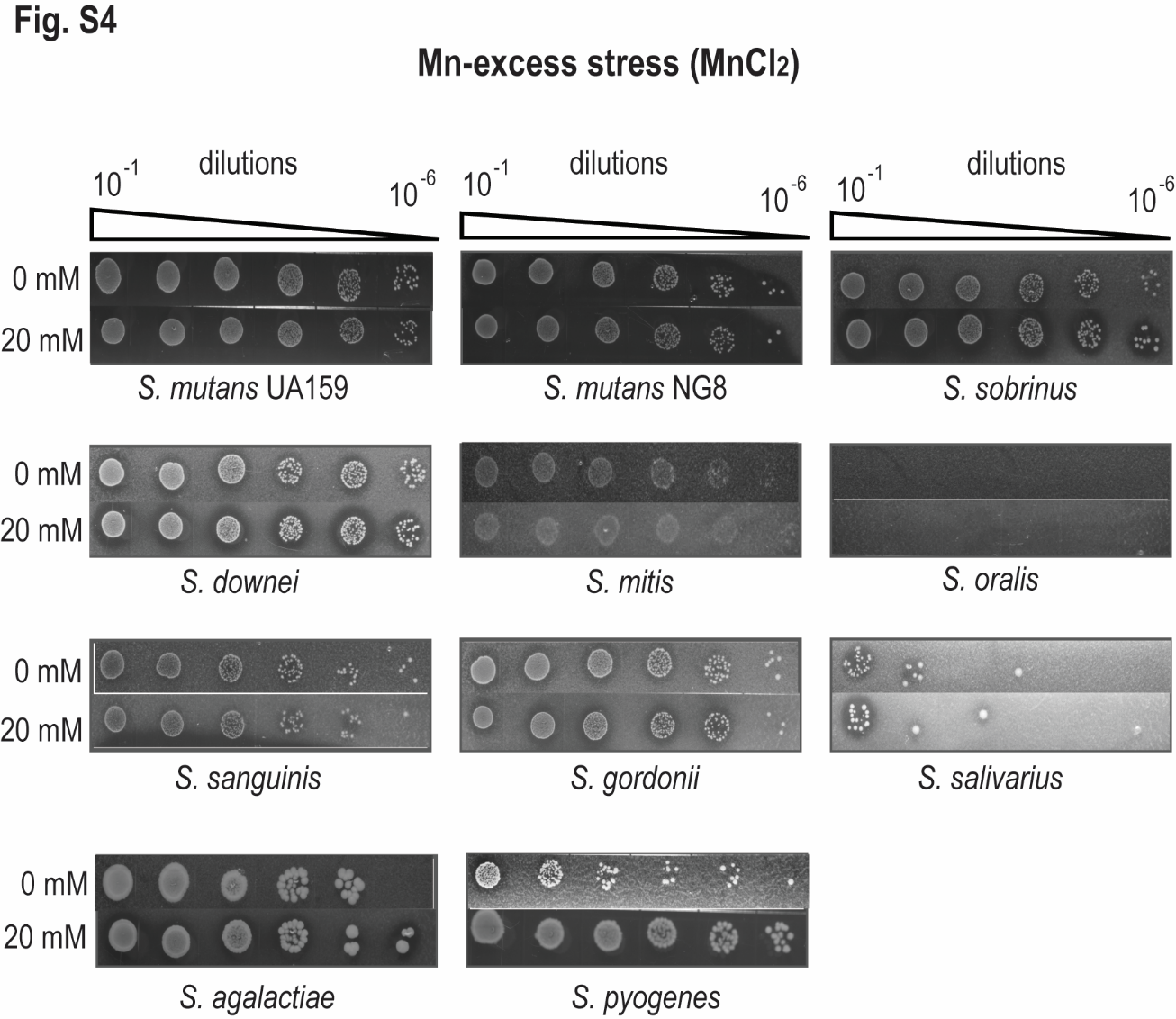


**Fig. S4: Impact of MgCl2 supplementation on the survival of Streptococcus spp. when exposed to 5 mM MnCl2.** Overnight cultures of *Streptococcus spp.* were diluted in BHI to an O.D._600_ of 0.2, followed by 10-fold serial dilutions in BHI. Drops (4 μL) were spotted onto BHI plates containing 5 mM MnCl_2_ ± 20 mM MgCl_2_, ranging from the 10^-1^ dilution (left) to the 10^-6^ dilution (right). Plates were incubated at 37 °C in a 5% CO_2_ atmosphere for 2 days before documentation. The images shown are representative of three independent experiments.


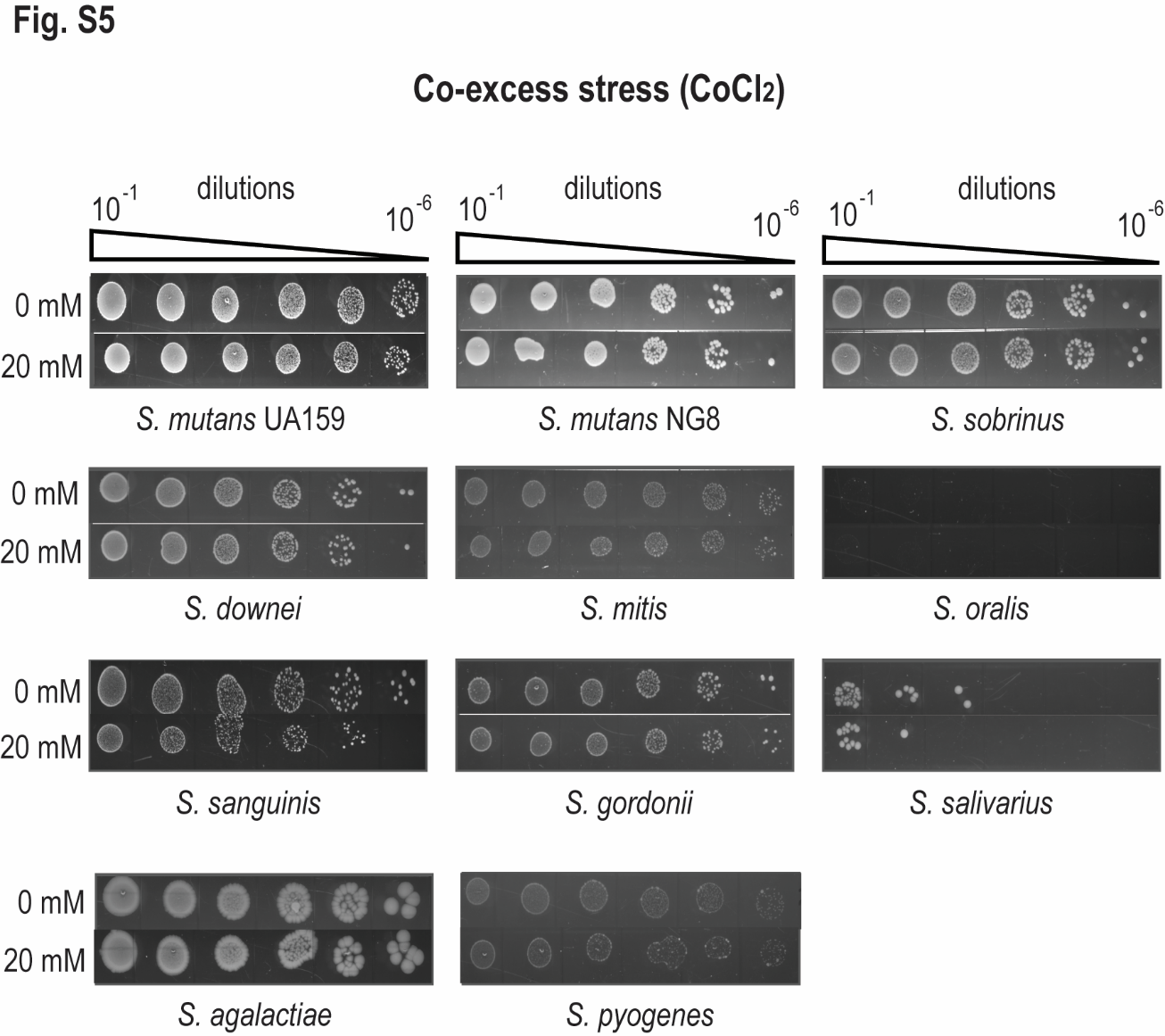


**Fig. S5: MgCl_2_ supplementation promotes the survival of various *Streptococcus spp.* when exposed to 5 mM CoCl_2_.** Overnight cultures of *Streptococcus spp.* were diluted in BHI to an O.D._600_ of 0.2, followed by 10-fold serial dilutions in BHI. Drops (4 μL) were spotted onto BHI plates containing 1.5 mM CoCl_2_ ± 20 mM MgCl_2_, ranging from the 10^-1^ dilution (left) to the 10^-6^ dilution (right). Plates were incubated at 37 °C in a 5% CO_2_ atmosphere for 2 days before documentation. The images shown are representative of three independent experiments.

CLUSTAL 2.1 multiple sequence alignment

Smi ------------MEDPSSQNLLLQFVLLFILTVLNAFFSATEMAMVSLNRARVEQKAEEG

Sor ------------MEDPSSQNLLLQFVLLFILTFLNAFFSATEMAMVSLNRARVEQKAEEG

Ssa ------------MEDPGSQNMLWQALLLFILTLLNAFFSAAEMAMVSLNRARVEQKAEEG

Sgo ------------MEDPSSQTLLLQFLLLVVLTFLNAFFSAAEMAMVSLNRARVEQKAEEG

San ------------MEDPSSQTLLLQALLLLVLTLLNAFFSAAEMAMVSLNRARVEQKAEEG

Spy ------------MEDPVSQPLVIQFLLLVVLTLLNAFFSASEMALVSLNRSRVEQKAADG

Sag ------------MEDPGSQSLLLQFVILLILTLFNAFFSASEMALVSLNRSKVEQKAEEG

Sdo ------------MEDPSSQNLLFQMLLLVVLTCLNAFFAASEMSMVSLNRSRVEQKAEEG

Sso MKAIYNFRRKNLMEDPSSQNLLFQILLLIILTVLNAFFAASEMSLVSLNRARVEQKAEEG

Scr ------------MEDPSSQHLLFQLLLLVVLTSLNAFFAASEMSMVSLNRARVEQKAEEG

Smu ------------MEDPGSQSLILQFLLLLILTLCNAFFSATEMALVSLNRARVEQKAEEG

Sra ------------MEDPGSQSLILQFLILLILTLCNAFFSATEMALVSLNRARVEQKAEEG

Sal ------------MEDSSGQSLLFQSILLLILTFLTAFFSASEMALVSLNRSRVEQKAEEG

Sth ------------MEDSSGQSLLFQFILLLILTLLSAFFSASEMALVSLNRSRVEQKAEEG

MpfA -------------------MDIVNLILVAVLIALTAFFVASEFAIIRIRGSRIDQLIAEG

MpfB -----------------------MIIAIIILIFISFFFSGSETALTAANKTKFKTEADKG

: : :* . ** .:* :: . ::.. .*

Smi DRRYIRLLKVLENPNHFLSTIQVGITLITILSGASLAETLGREIASWLGNG----ETSYA

Sor DKRYIRLLKVLENPNHFLSTIQVGITLITILSGAKLADTLGQVIASWMGSG----ETAYA

Ssa DLKYIRLLAVLESPNNFLSTIQVGITVINILSGASFADNLGKLFSSWMGNS----ETARA

Sgo DIKYIRLLQVLENPNNFLSTIQVGITLINILSGASFADTLGRVFSSLMGNS----KTAHA

San DAKYIRLLKVLENPNHFLSTIQVGITLITILSGASLANTLGQEIASWMGNS----ETARA

Spy DKKYARLLRVLEEPNHFLSTIQVGITFISLLSGASLSASLGKVISGWLGNS----ATART

Sag DKRYRRLLDVLENPNNFLSTIQVGITFISLLQGASLSASLGHVISGWLGNS----ATART

Sdo DKKYIRLVKVLDKPNNFLSTIQVGITFISLLQGASLSASLGKVIAGWFGNS----ATAQT

Sso DKKYIRLVKVLDKPNNFLSTIQVGITFISLLQGASLSASLGKVIASWFGNS----ATAQT

Scr DKKYIRLVKVLDKPNNFLSTIQVGITFISLLQGASLSASLGKVIAGWFGNS----AAAQT

Smu EKKYIRLLKVLENPNNFLSTIQVGITLITLLSGASLADSLGREIAVWFGNS----ATART

Sra DKKYIRLLKVLEKPNHFLSTIQVGITLITLLSGASLADSLGGEIAAWFGNS----ATART

Sal DKKFIRLLKVLENPNNFLSTIQVGITFISLLQGASLSASLGAVIATWFGHA----AWAKT

Sth DKKFIRLLKVLENPNNFLSTIQVGITFIGLLQGASLSASLGAVIATWFGNV----AWAKT

MpfA NKAAIAVKKVTTHLDEYLSACQLGITLTSIGLGVLGESTIERLLHPLFVQMNVPGSLSHV

MpfB DKKAKGIVKLLEKPSEFITTILIGNNVANILLPTLVTIMALRWGISVGIAS---------

: : : ..:::: :* .. : .

Smi VASFLSLAFLTYISIVFGELYPKRIALNLKDALAIRTAPVIIGLGKLVSPFVWLLSASTN

Sor IASFLSLAFLTYISIVFGELYPKRIALNLKDNLAIRTAPVIIGLGKIVSPFVWLLSASTN

Ssa IGTFLALVLLTYISIVLGELYPKRIAMNLKDNLAVRAAPVIIFLGKIVSPFVWLLSASTN

Sgo IASFLALALLTYVSIVFGELYPKRIALNLKDDLAVRAAPFIIFLGKLVSPFVWLLSASTN

San IASFLSVAILTYISIVFGELYPKRIALNLKDSLAVRTAPVIIFLGKIVSPFVWLLSASTN

Spy AGTIISLVFLTYVSIVLGELYPKRIAMNLKDKLAIVSAPIIIGLGRLVSPFVWLLSASTN

Sag AGSIIALIFLTYVSIVLGELYPKRIAMNLKDRLAIVSAPIIIFLGKIVSPFVWLLSASTN

Sdo AGSIISLIFLTYIAIVLGELYPKRIAMNLKDNFAIYSAPVIVGLGKVVSPFVWFLSASTN

Sso AGSIISLIFLTYIAIVLGELYPKRIAMNLKDNFAIYSAPVIVGLGKVVSPFVWFLSASTN

Scr AGSIISLIFLTYIAIVLGELYPKRIAMNLKDNLAIYSAPVIVGLGKIVSPFVWFLSASTN

Smu AGSLISLAFLTYISIVLGELYPKRIAMNLKENLAVLSAPVIIFLGKVVSPFVWLLSVSTN

Sra AGSIIALAFLTYISIVLGELYPKRIAMNLKDSLAVYSAPLIILLGKLVSPFVWLLSASTN

Sal AGSMISLVVLTYISIVFGELYPKRIAMNLKENLAIYSAPVIIVTGKIVSPFVWILSASTN

Sth AGSVVSLVVLTYISIVFGELYPKRIAMNLKENLAIYSAPVIIVTGKIVSPFVWILSASTN

MpfA ISFIFAYAIITFLHVVVGELAPKTVAIQKAEAVSMLFAKPLIWFYRIAFPFIWLLNNSAR

MpfB -------AVLTVVIILISEVIPKSVAATFPDKITRLVYPIINICVIVFRPITLLLNKLTD

.:* : ::..*: ** :* : .: : : *: :*. :

Smi LLSNLT-PMTFDDADEKMTRDEIEYMLTNSEE--TLDAEEIEMLQGIFSLDELMAREVMV

Sor LLSRVT-PMTFDDADEKMTRDEIEYMLTKSEE--TLDADEIEMLQGIFSLDELMAREVMV

Ssa LLSRIT-PMKFDDADEKMTRDEIEYMLTNSEE--TLDADEIEMLQGIFSLDELMARELMV

Sgo LLSRIT-PMKFDDADEKMTRDEIEYMLTKSEE--TLDADEIEMLQGIFSLDELMARELMV

San LLSRIT-PMTFDDADEKMTRDEIEYMLTNSEE--TLDADEIEMLQGIFSLDEMMAREVMV

Spy LLSRLT-PMTFDDADEQMTRDEIEYMLSKSEA--TLDAEEIEMLQGVFSLDEMMAREVMV

Sag LLSRIT-PMTFDDADEKMTRDEIEYMLTNSEE--TLDAEEIEMLQGIFSLDEMMAREVMV

Sdo LLSRIT-PMTFDDADEKMTRDEIEYMLTNSEE--TLEQDEIEMLQGVFSLDELMAREVMV

Sso LLSRMT-PMTFDDADEKMTRDEIEYMLTNSEE--TLEQDEIEMLQGVFSLDELMAREVMV

Scr LLSRIT-PMTFDDADEKMTRDEIEYMLTNSEE--TLDQEEIEMLQGVFSLDELMAREVMV

Smu LLSRLT-PMTFDDADEKMTRDEIEYMLTNSEE--TLDADEIEMLQGVFSLDELMAREVMV

Sra LLSRIT-PMTFDDADEKMTRDEIEYMLTNSEE--TLDADEIEMLQGVFSLDELMAREVMV

Sal LVSHLT-PMTFDDADEQMTRDEIEYMLAKSED--TLEAEEIEMLQGIFSLDELMAREVMV

Sth LLSRLT-PMTFDDADEPMTRDEIEYMLTKSED--TLEAEEIEMLQGIFSLDELMAREVMV

MpfA LLTKAFGLETVSENELAHSEEELRIILSESYKSGEINQSEFKYVNKIFEFDDRLAKEIMI

MpfB SINRSL--SKGQPQEHQFSKEEFKTMLAIAGHEGALNEIETSRLEGVINFENLKVKDVDT

:.. . . : :.:*:. :*: : :: * . :: ::.::: .:::

Smi P-RTDAFMVDIQDDSQTIIQSILKQNYSRIPVYDGDKDNVIGIIHTKSLLNAGFV-DGFD

Sor P-RTDAFMVDIQDDSQTIIQSILKQNFSRIPVYDGDKDNVIGLIHTKRLLNAAYA-DGFE

Ssa P-RTDAFMVDIQDDTKEIIESILKQSFSRIPVYDGDKDNVIGLIHTKRLLNEGFI-NGFD

Sgo P-RTDAFMVDIQDDTKEIIESILKQSFSRIPVYDGDKDNVIGLIHTKRLLNEGFV-NGFD

San P-RTDAFMVDINDDTKEIIESILKQNFSRIPVYDDDKDNVIGLIHTKRLLNEAFT-NGFD

Spy P-RTDAFMIDINDDPLENIQEILKQSFSRIPVYDVDKDKIIGLIHTKRLLESGFR-QGFD

Sag P-RTDAFMIDINNDAQSNIEGILSQNFSRVPVYDDDKDRVVGVLHTKRLLEVGFK-TGFD

Sdo H-RTDAFMVDINDDPQEIIKEILKKNFSRIPVYDDDKDKVIGLIHTKKLLAAGFE-NGFE

Sso H-RTDAFMVDINDDPQEIIKEILKKNFSRIPVYDDDKDKVLGLIHTKKLLEVGFN-NGFE

Scr H-RTDAFMVDINDDPQEIIKEILKKSYSRIPVYDDDKDKVLGLIHTKKLLEVGFN-NGFE

Smu P-RTDAFMVDINDDSSDIIQTILNERFSRIPVYDDDKDKIIGIIHTKNLLNAGFK-EGFD

Sra P-RTDAFMVDINDDTSDIIQAILNESFSRIPVYDGDKDKIIGLIHTKRLLDAGFK-EGFD

Sal P-RTDAFMIDIEDNTQENIQAILKESFSRIPVYEDDKDKIIGVIHTKNLLKAGFE-LGFE

Sth P-RTDAFMIDIEDDTQENIQAILKQSFSRIPVYEDNKDKIIGVIHTKKLLKAAYE-LGFE

MpfA P-RTEIVSLPHDIKISEMMDIIQIEKYTRYPVEEGDKDNIIGVINIKEVLTACISGEVSV

MpfB TPRINVTAFASNATYEEVYETVMNKPYTRYPVYEGDIDNIIGVFHSKYLLAWSNK----K

* : . : . : : ::* ** : : *.::*::: * :*

Smi NIVWKKILQDPLFVPETIFVDDLLKELRNTQRQMAILLDEYGGMAGLVTLEDLLEEIVG-

Sor NIVWKKILQDPLFVPETIFVDDLLKELRNTQRQMAILLDEYGGMAGLVTLEDLLEEIVG-

Ssa NIVLRKILQEPLFVPETMFVDDLLKELRNTQNQMAILLDEYGGMAGLVTLEDLLEEIVG-

Sgo NIVLRKILQEPLFVPETMFVDDLLKELRNTQNQMAILLDEYGGMAGLVTLEDLLEEIVG-

San NIVLRKILQEPLFVPETIFVDDLLTELRNTQNQMAILLDEYGGMSGLVTLEDLLEEIVG-

Spy QINMRKMLQEPLFVPETIFVDDLLRQLRNTQNQMAILLDEYGGVAGLVTLEDLLEEIVG-

Sag TIDLRKILQEPLFVPETIFVDDLLKALRNTQNQMAILLDEYGGVAGLVTLEDLLEEIVG-

Sdo NLNLRRILQEPLFVPETIFIDDLLKDLRRTQNQMAILLDEYGGVAGLVTLEDLLEEIVG-

Sso NISLRRILQEPLFVPETIFVDDLLKELRRTQNQMAILLDEYGGVSGLVTLEDLLEEIVG-

Scr TINLRRILQEPLFVPETIFVDDLLKELRRTQNQMAILLDEYGGVAGLVTLEDLLEEIVG-

Smu HINLRRILQEPLFVPETIVVNDLLTALKNTQNQMAILLDEYGGVAGLVTLEDLLEEIVG-

Sra HINLRRILQEPLFVPETIFVNDLLTALRNTQNQMAILLDEYGGVAGLVTLEDLLEEIVG-

Sal NIKLRRIMNEPLFVPETIFVDDLLADFRNTNNQMAILLDEYGGVAGLVTFEDLLEEIVG-

Sth NVNLRRIMNEPLFVPETIFVDDLLAAFRNTNNQMAILLDEYGGVAGLVTFEDLLEEIVG-

MpfA DSTISQFVNPIIHVIESAPIQDLLVKMQKERVHMAILSDEYGGTAGLVTVEDIIEEIVG-

MpfB EDQITNYSAKPLFVNEHNKAEWVLRKMTISRKHLAIVLDEFGGTEAIVSHEDLIEELLGM

. :.* * : :* : . ::**: **:** .:*: **::**::*

Smi EIDDETDKTEI-EVHKIGEDTYIVQGTMNLNDFNDYFDVELESDDVDTIAGYYLTGVGTI

Sor EIDDETDRAEI-EVHQIGEDTYIAQGTMNLNDFNNYFGVELESDDVDTIAGYYLTGVGTI

Ssa EIDDETDKAEI-EVHEIGENTYIVLGTMTLNDFNEYFEVEIESDDVDTIAGYYLTCVGTI

Sgo EIDDETDKAEI-EVHEIGENTYIVIGTMTLNDFNEYFEVEIESDDVDTIAGYYLTGVGTI

San EIDDETDKAEV-EVYQVSDHIYFVLGTMSLNDFNEYFEVELESDDVDTIAGYYLTGVGSI

Spy EIDDETDKAEQ-FVHEIGDNTYIVVGTMTLNEFNDYFDTELESDDVDTIAGFYLTGIGTI

Sag EIDDETDTAEQ-FVREIDENIYIVLGTMTLNEFNDYFETELESDDVDTIAGYYLTGVGSI

Sdo EIDDETDQAEV-EVREIDQDTYIVLGTMTLNDFNEYFETELESDDVDTIAGYYLTGVGNI

Sso EIDDETDQAEV-EVREIDKDTYIVLGTMTLNDFNEHFETELESDDVDTIAGYYLTGVGNI

Scr EIDDETDQAEV-EVREIAPDTYIVLGTMTLNDFNEYFETELESDDVDTIAGYYLTGVGNI

Smu EIDDETDKTAI-SVREIADNTYIVLGTMTLNDFNEYFETDLESDNVDTIAGFYLTGVGTI

Sra EIDDETDRTSI-SVREIAENTYIVLGTMTLNDFNEYFETNLESDDVDTIAGFYLTGVGTI

Sal EIYDETDKSSV-EVREIGENTYIVEGAMTLNDFNEHFDTELESDDVDTIAGYYLTGVGAI

Sth EIDDETDKASV-EVREIGENTYIVEGSMTLNDFNEHFDTELESDDVDTIAGYYLTGVGAI

MpfA EIRDEFDIDEISEIRKIGEGHYILDGKVLIDQVNDLLGIHLENEEVDTIGGWFLTQKYDV

MpfB EIEDEMDKKEK-------------------------------------------------

** ** *

Smi P-TTEKLSYELVSQNKQLILTNDKVKNGRVTKVKVQITEVEIEEETE--

Sor P-TTEKISYELVSQNKQIVLTNDKVKNGRVTKVKVQITELEPEEETE--

Ssa PDPKERISYEVESQNKQLILTNDKVKNGRVTKVKVEISEQVEIDEEANK

Sgo PDPKERISFEVESQNKKLILTNDKVKNGRVTKVKVEISEQVEADEEVTK

San PDVKERLSYEVESQGKKLILTNDKVKNGRVTKLKVEVSEIVEEEEEVEK

Spy PSQEQKEAYEIDNKDKHLVLINDKVKDGRITKLKLILSNIEQIIEED--

Sag PNQEEKVAYEVDCKDKHITLINDKVKDGRITKLKVLLSDIEQNIEKD--

Sdo PSQEEKESFQVDSGKHHLEIINDKVKEGRVTKVKVILSPLEEAEADKE-

Sso PSQEEKESFEVDSGKHHLEIINDKVKEGRVIKVKVILSPLEEVEEEKE-

Scr PSQEEKESFHVDSGQRHLEIINDKVKEGRVTKVKVLVSPLEEADDVKE-

Smu PSQEEKEHFEVESNGKHLELINDKVKDGRVTKLKILVSEVEEKEDEKD-

Sra PSQDEKEHFTVDSNNKHLELINDKVKDGRITKLKLLVTEIEETEDEKD-

Sal PTQEVKEHYSVINKDKHLEFINDKVKDGRVTKLKVIITAAPEEAEE---

Sth PTQEVKEHYEVINKDKRLEFINDKVKDGRVTKLKVIITAAPEEAGE---

MpfA EKDDSIIEEGCEFIINEIDGHHVAYIEVKKLQEEELLETANQQEA----

MpfB ----------EKLSQQQIQFQQRKNRNVSI-------------------

..: : :

**Fig. S6: Multiple sequence alignment showing the conservation of residues in HlyX homologs among various *Streptococcus spp.* (Sdo: *S. downei*; Sso: *S. sobrinus*; Scr: *S. criceti*; Sal: *S. salivarius*; Sth: *S. thermophilus*; Spy: *S. pyogenes*; Sag: *S. agalactiae*; Smu: *S. mutans*; Sra: *S. ratti*; Smi: *S. mitis*; Sor: *S. oralis*; Ssa: *S. sanguinis*; Sgo: *S. gordonii*; San: *S. sanguinis*; MpfA: *B. subtilis* MpfA; and MpfB: S. aureus MpfB).**


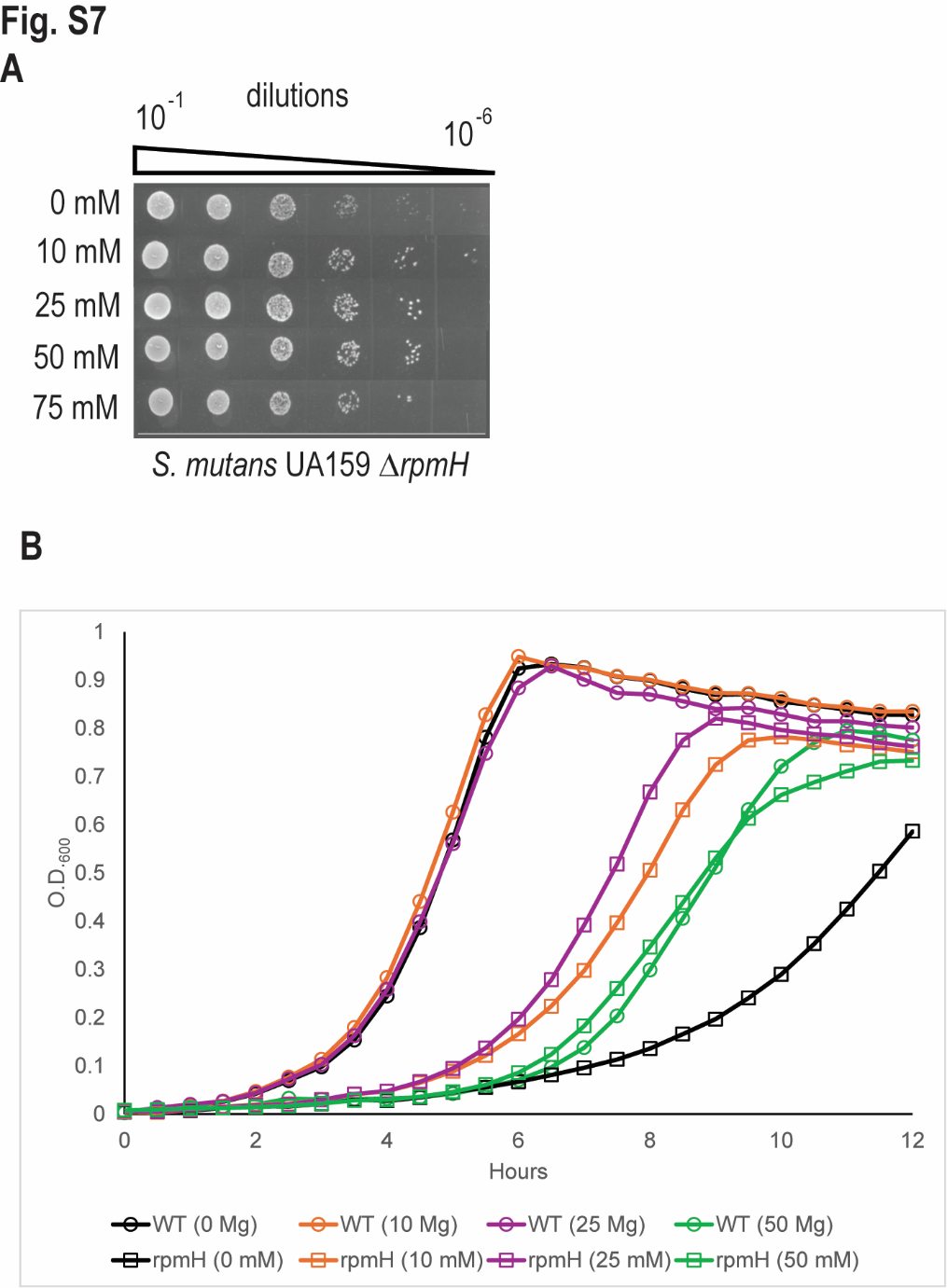


**Fig. S7: MgCl_2_ supplementation enhances the growth of *ΔrpmH* strain. A)** The EOP assay demonstrates the growth of *S. mutans ΔrpmH* on THYE supplemented with 0, 10, 25, 50, and 75 mM MgCl_2_. Overnight-grown cultures in THYE were diluted to an O.D._600_ of 0.2, followed by a 10-fold serial dilution in THYE. Drops (4 μL) were spotted onto a THYE plate from the 10^-1^ dilution (left) to the 10^-6^ dilution (right). Plates were incubated at 37 °C in a 5% CO_2_ atmosphere for 2 days before documentation. The images shown are representative of two independent experiments. B) Growth profile of *S. mutans* wild-type, *ΔrpmH* and complemented strain in THYE supplemented with 0, 10, 25, and 50 mM MgCl_2_. Overnight cultures were diluted 1:50 in THYE medium and grown to mid-log phase (OD_600_ ≈ 0.4), then diluted 1:100 into fresh THYE containing varying concentrations of MgCl₂. Growth was monitored using a Bioscreen C system in triplicate wells, with mineral oil overlaid to maintain anaerobic conditions.


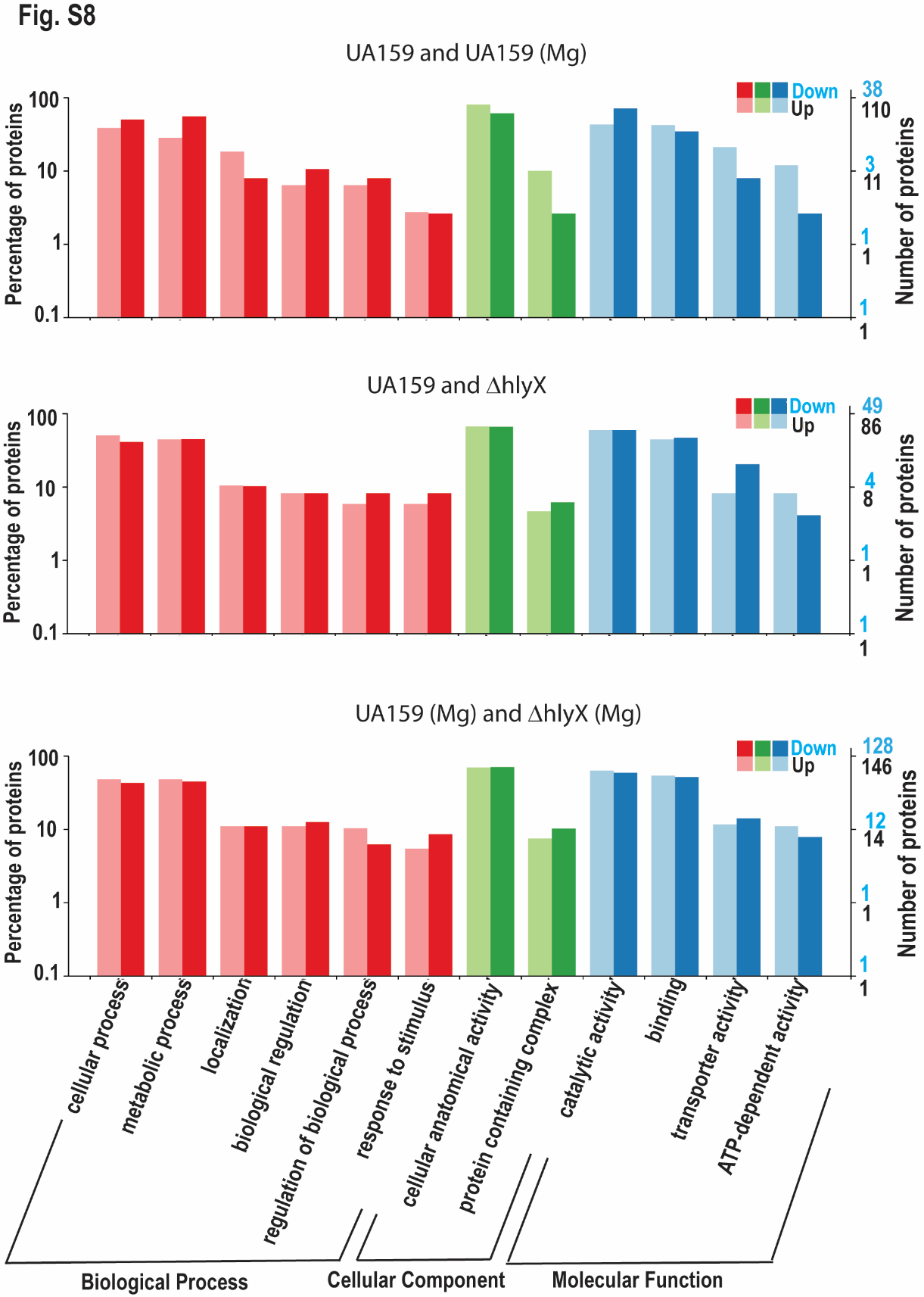


**Fig. S8: GO up and down analysis showing distribution of differentially expressed proteins (DEPs) annotated in GO level 2. A) MgCl_2_-treated to untreated wild-type, B) untreated *ΔhlyX* to untreated wild-type, and C) MgCl_2_-treated *ΔhlyX* to MgCl_2_-treated wild-type.** The x-axis represents GO term and y-axis represents the protein count. Dark colored bars represent the quantities of proteins decreased in abundance while light colored bars represent the quantities of proteins increased in abundance.


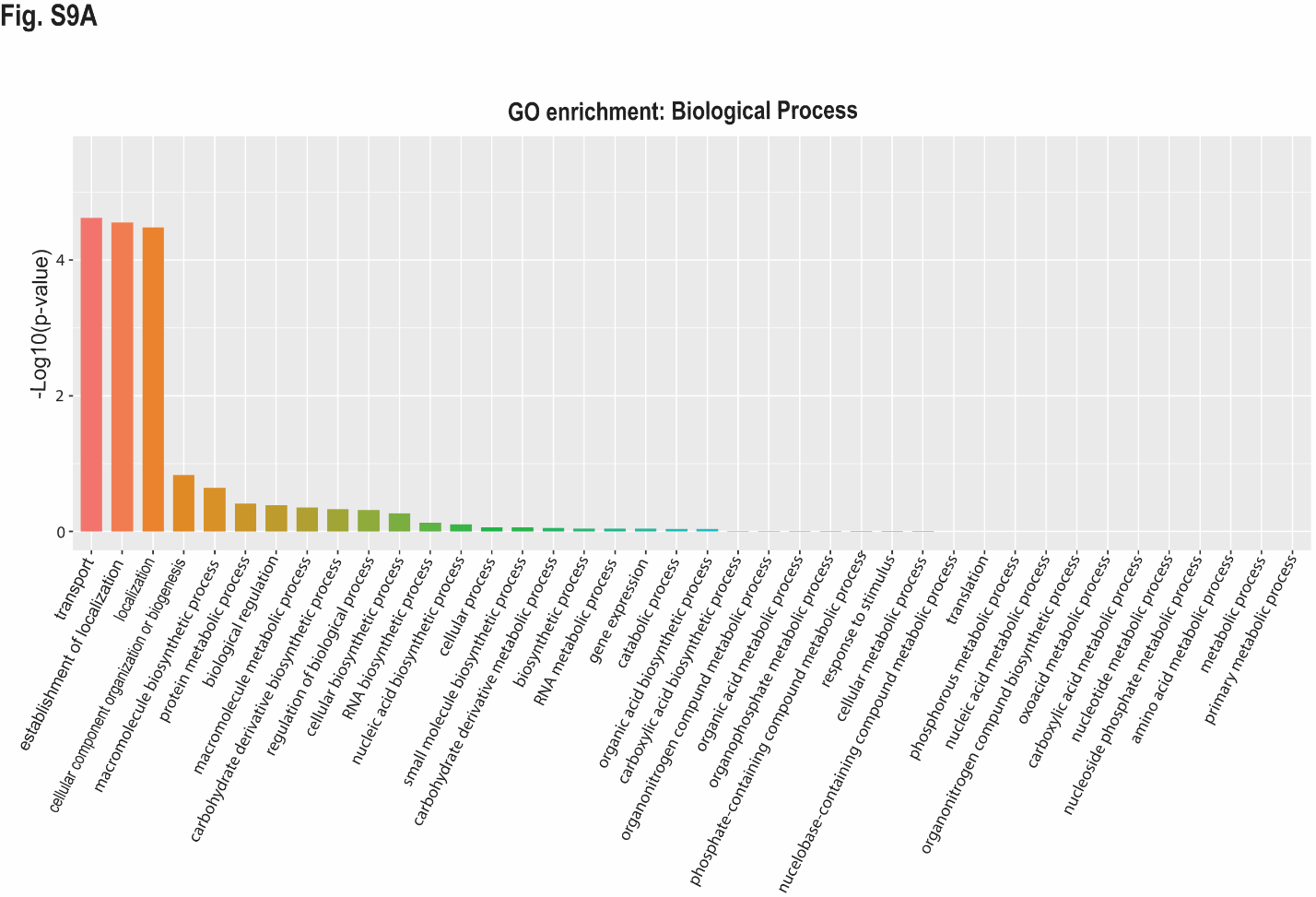

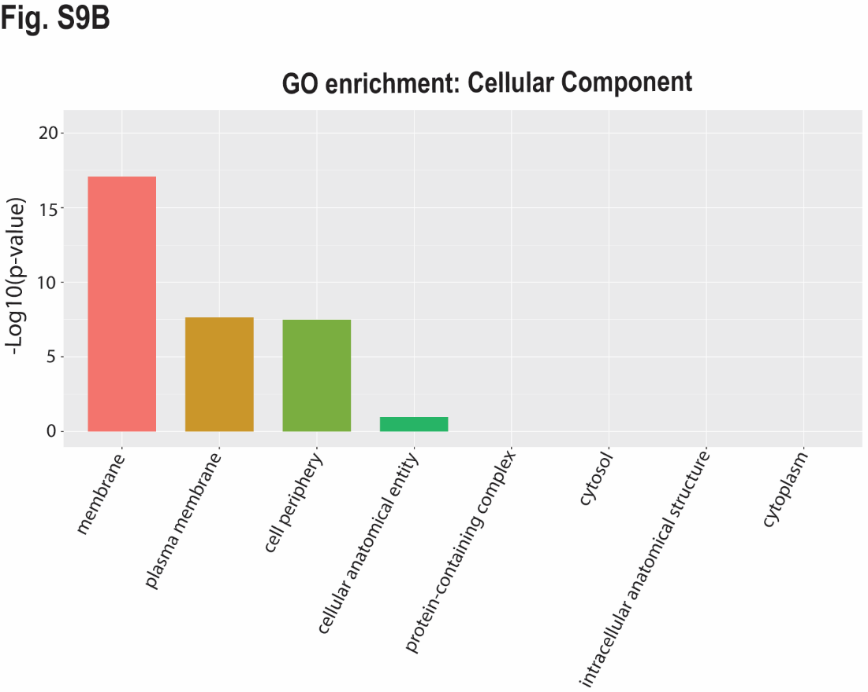

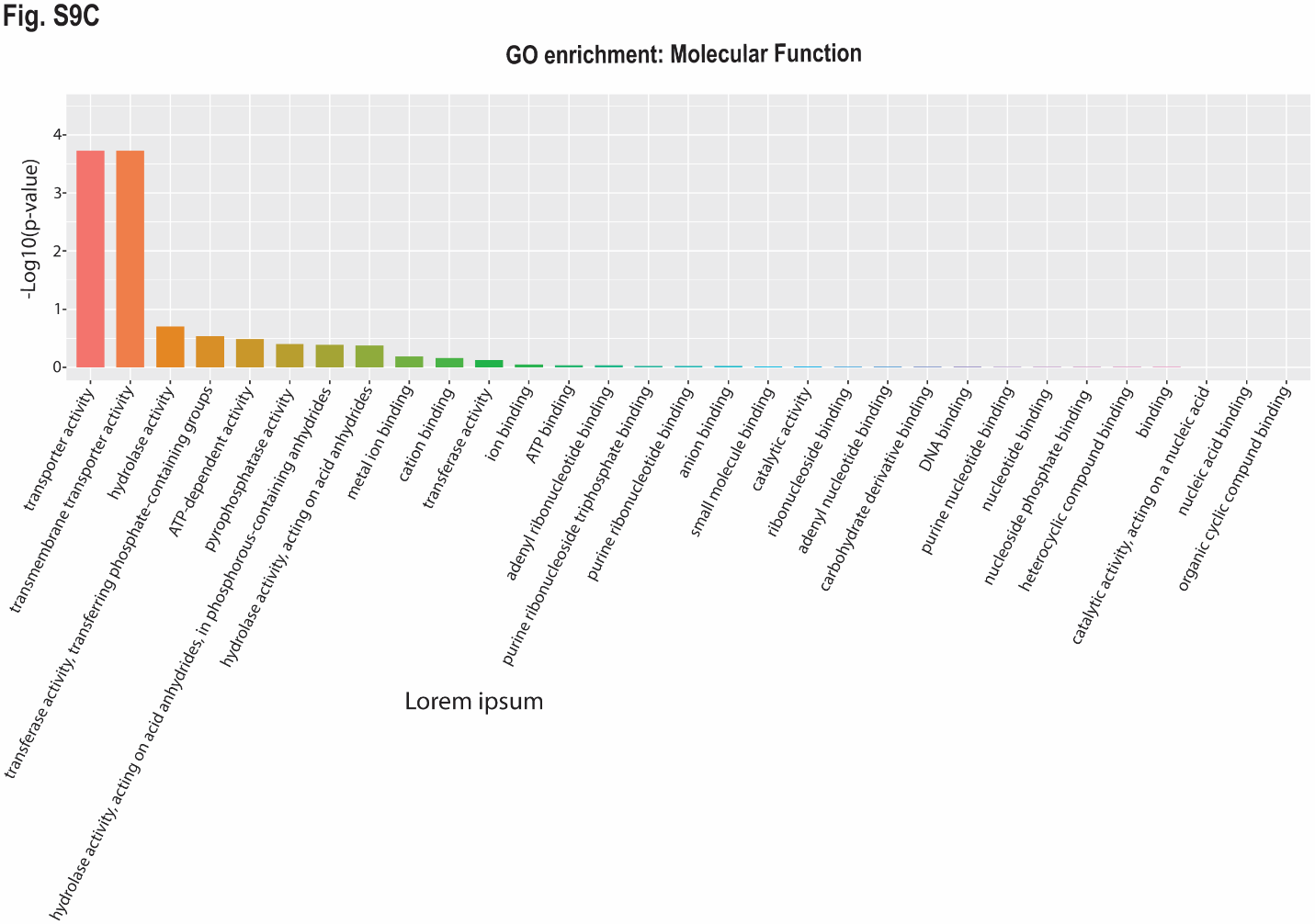


**Fig. S9: GO-based enrichment analysis of DEPs in the MgCl_2_-treated and untreated wild-type *S. mutans* cultures into GO functional categories. A) Biological processes, and B) Cellular component, and C) Molecular function (http://www.geneontology.org).**


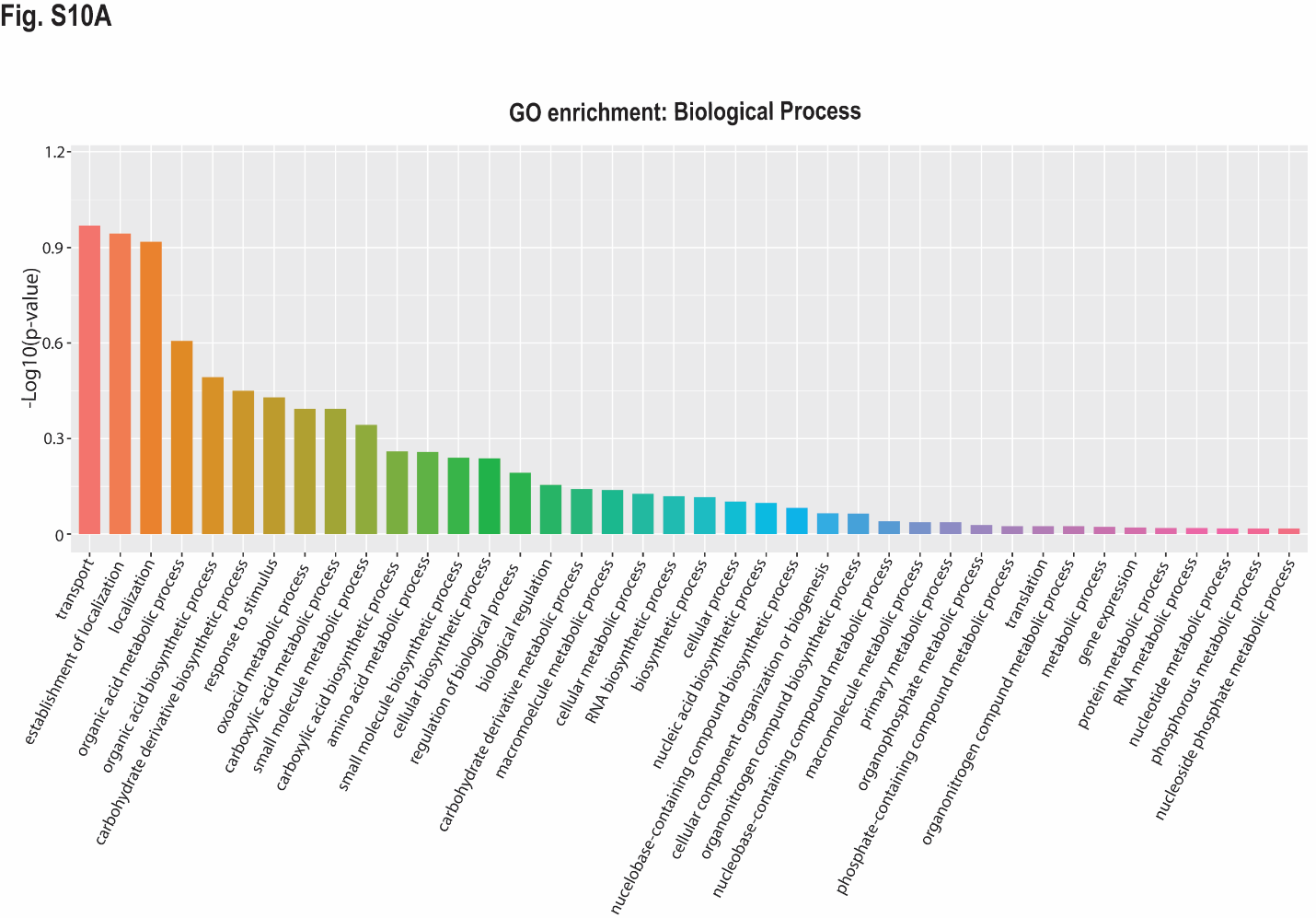


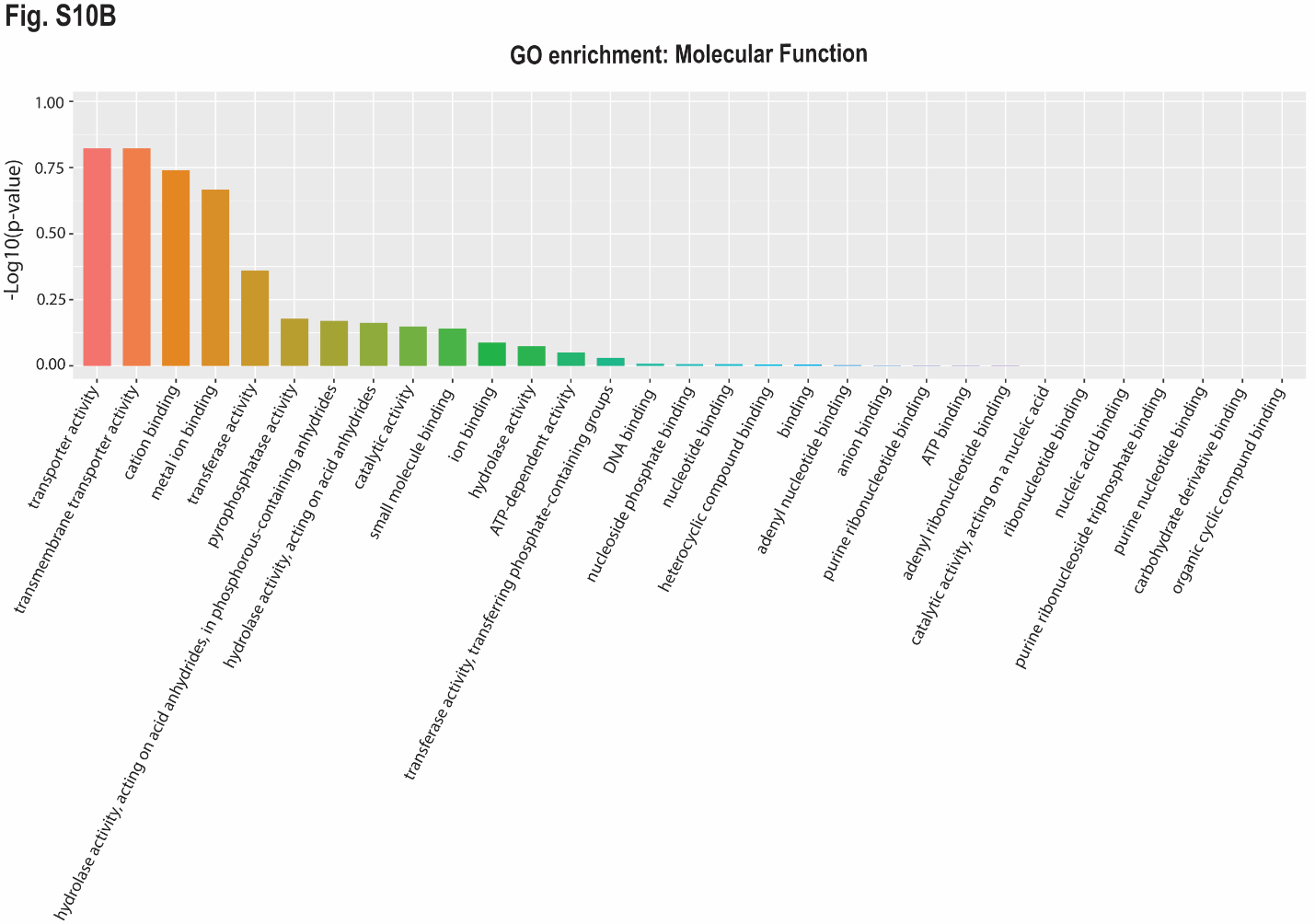


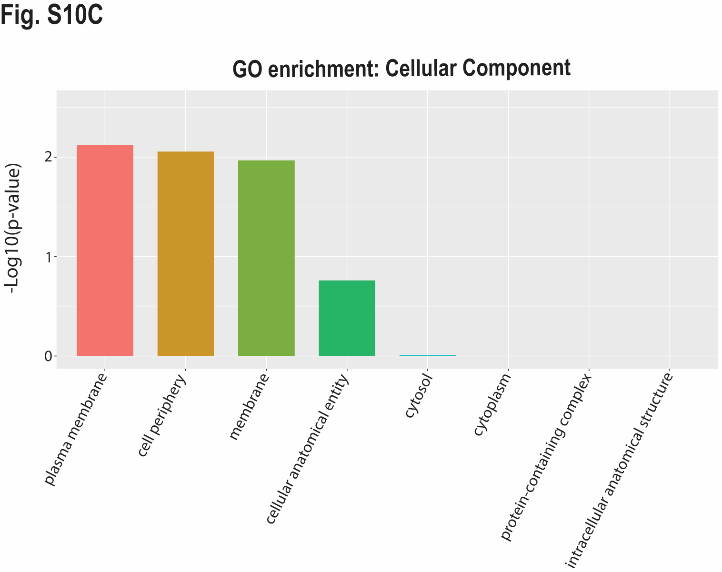


**Fig. S10:** **GO-based enrichment analysis of DEPs in the proteomes of wild-type and *ΔhlyX* strains into GO functional categories. A) Biological processes, B) Molecular function, and C) Cellular component (http://www.geneontology.org).**


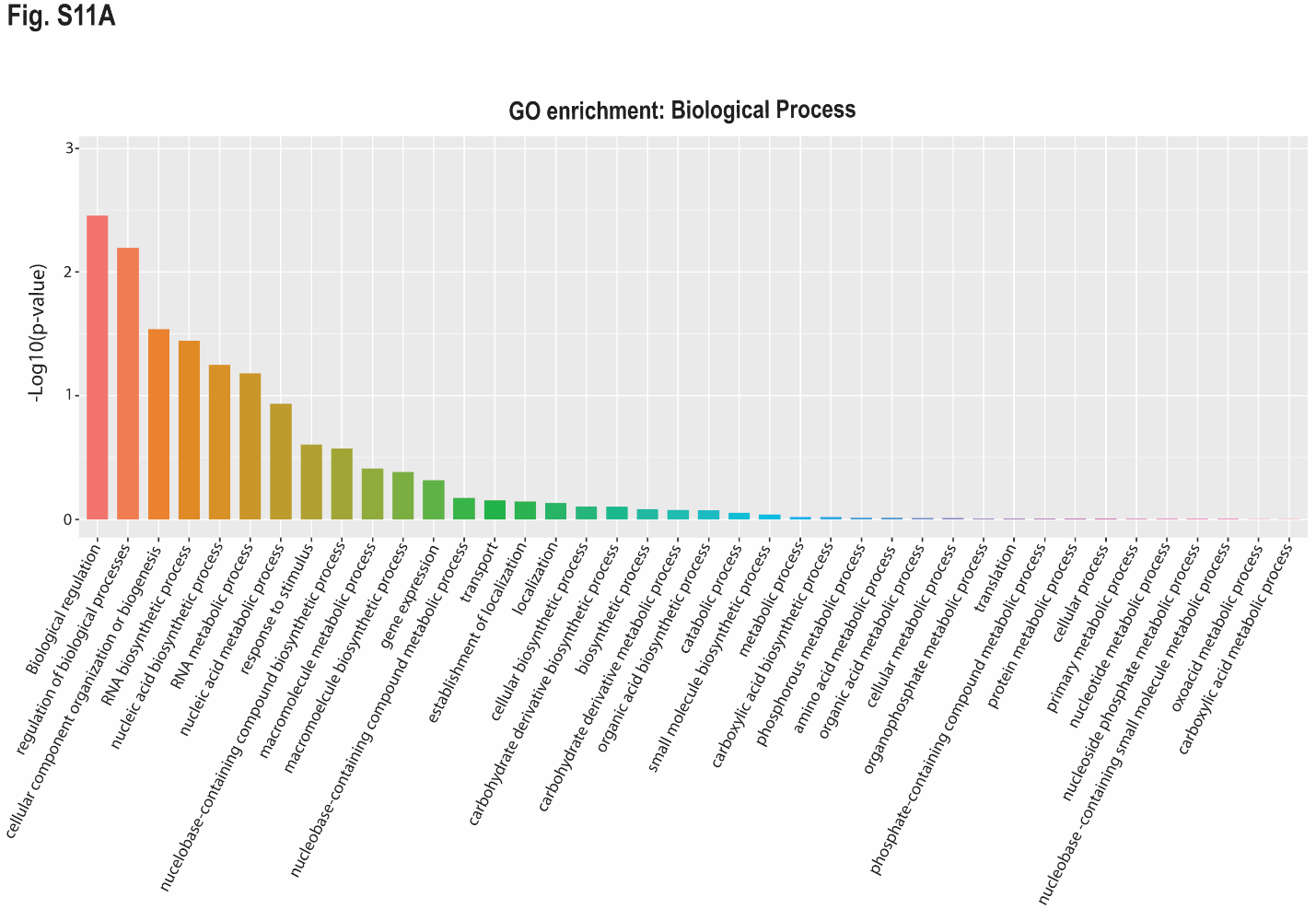


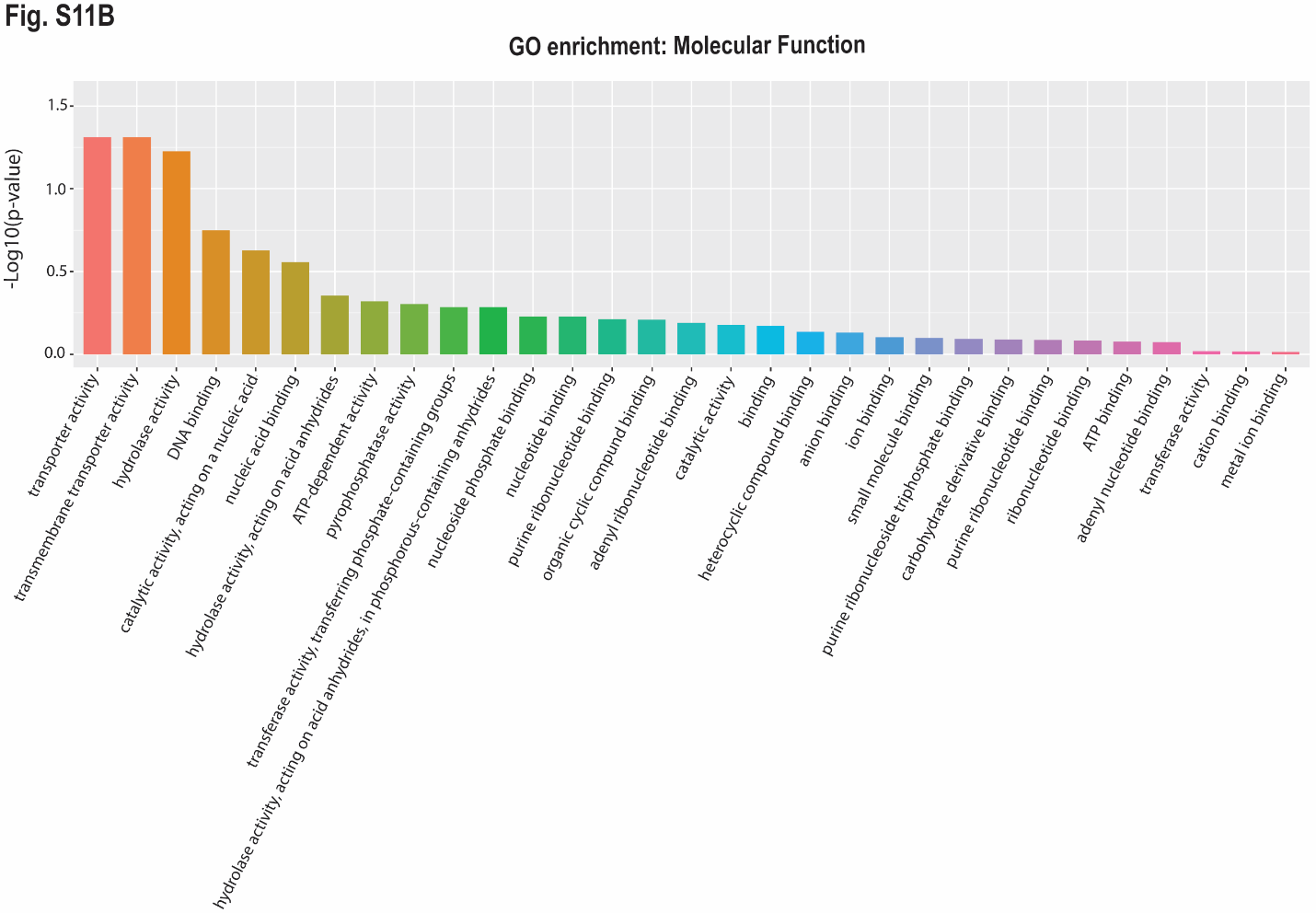

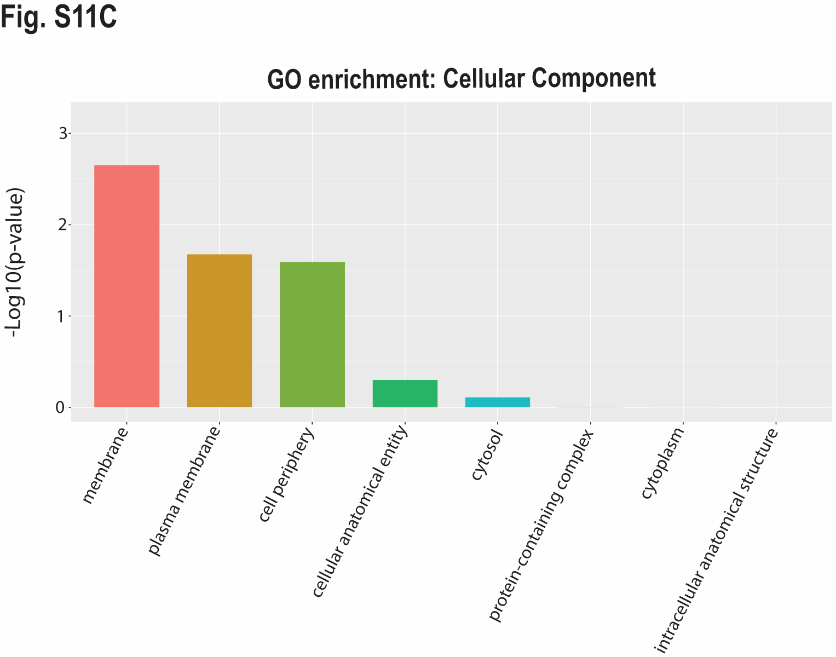


**Fig. S11: GO-based enrichment analysis of DEPs in the proteomes of MgCl_2_-treated wild-type and MgCl2-treated *ΔhlyX* strains into GO functional categories. A) Biological processes, B) Molecular function, and C) Cellular component (http://www.geneontology.org).**

**
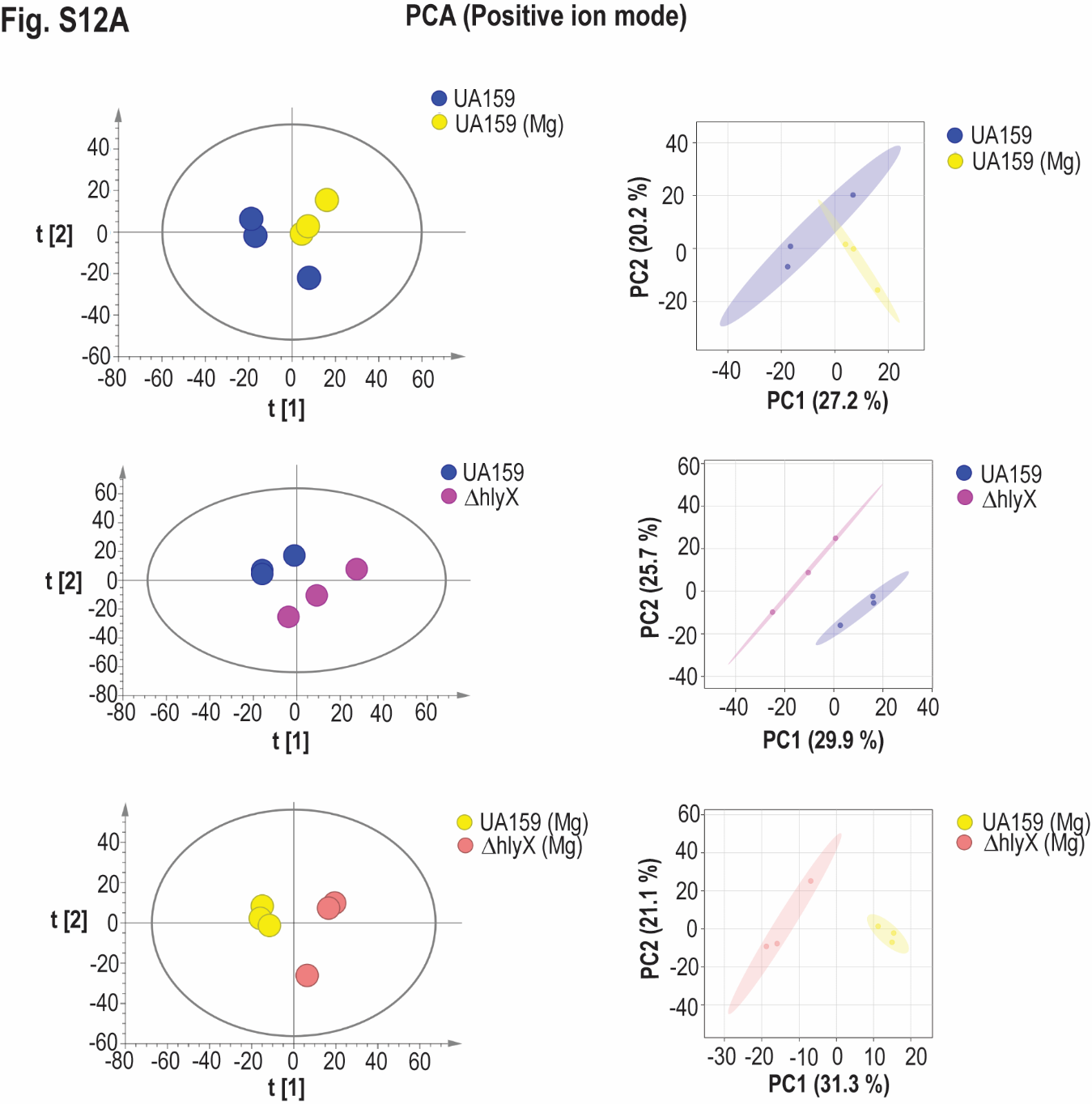

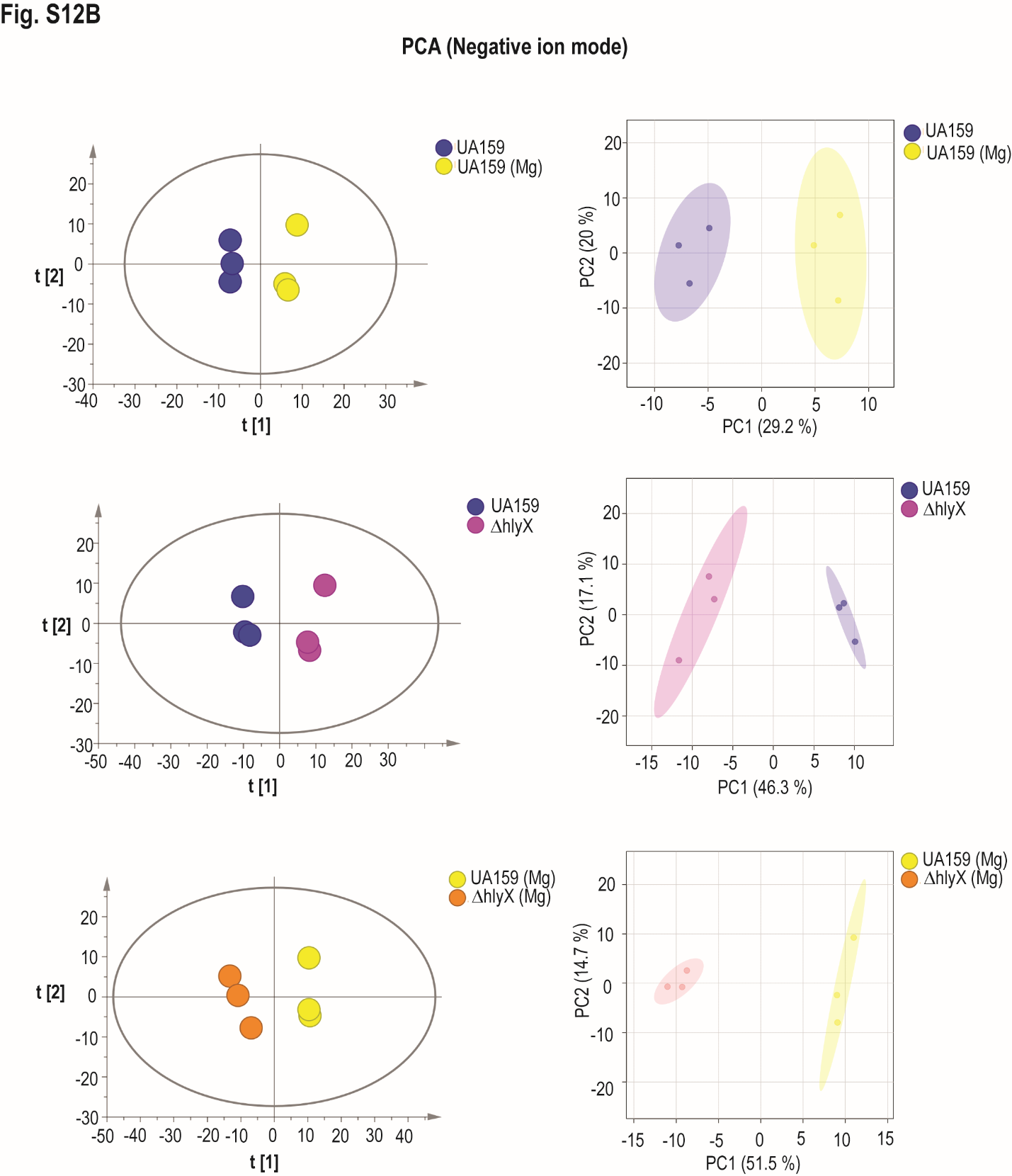

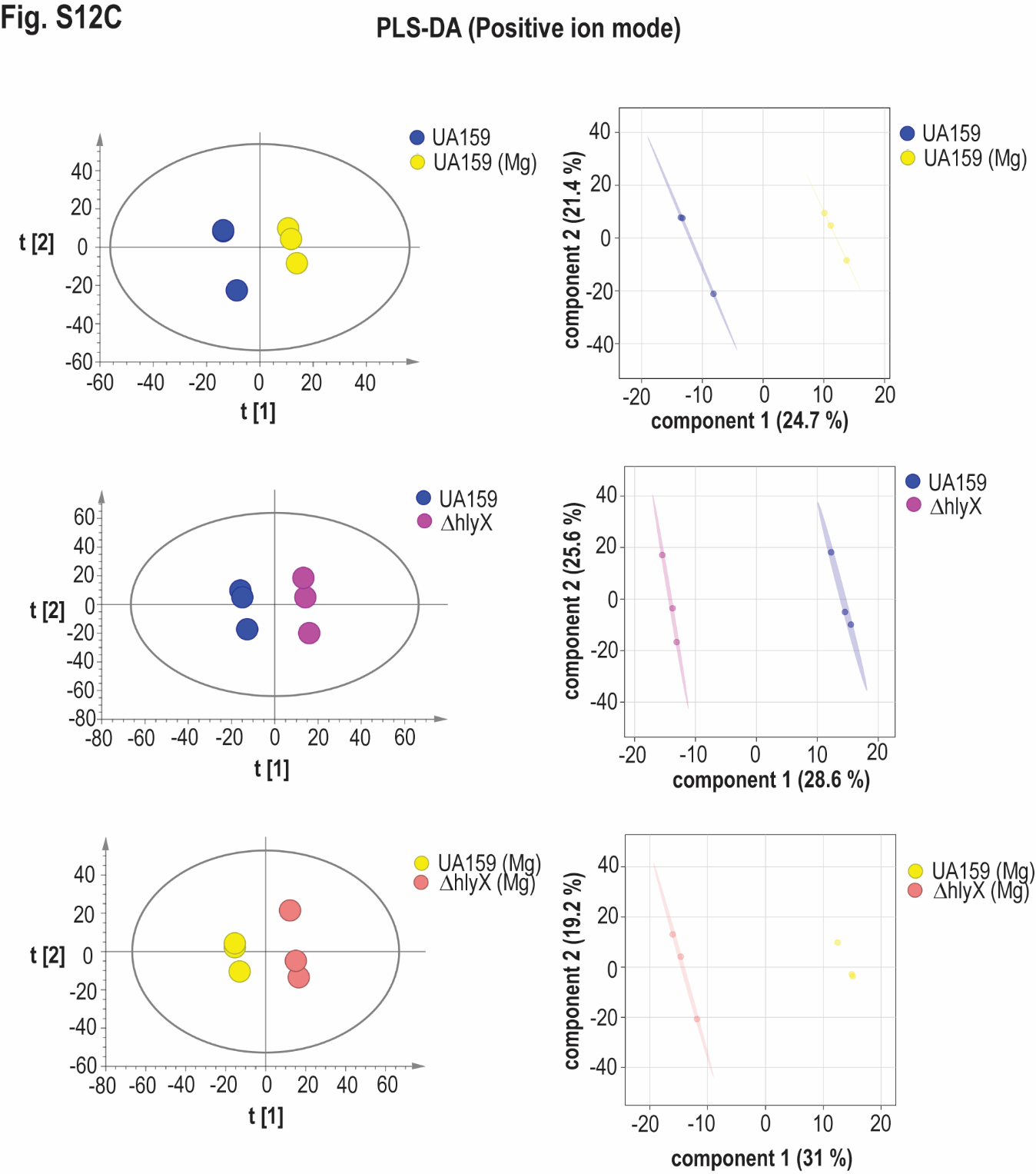

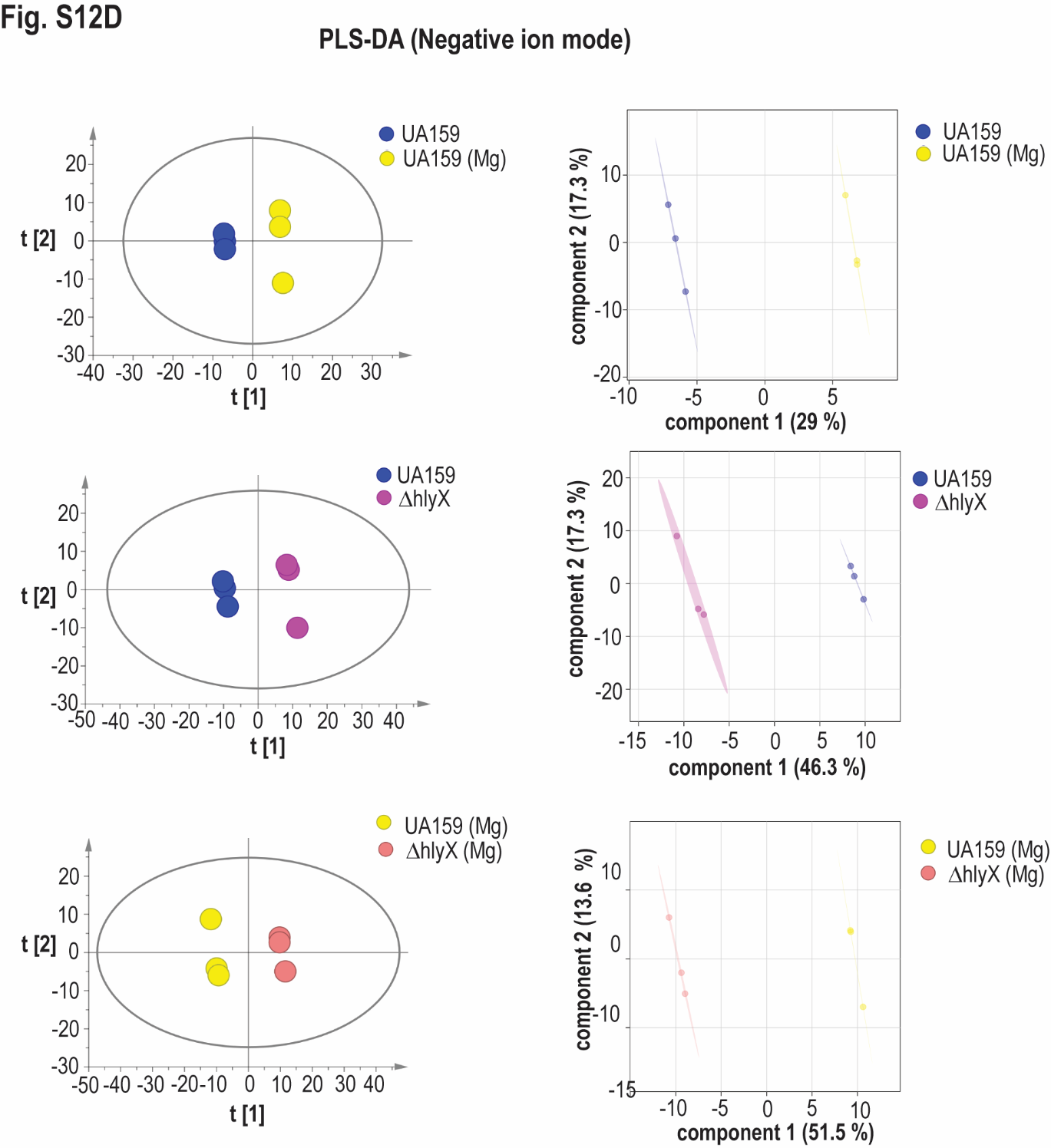
**

**Fig. S12: The Scores Scatter plot of A) PCA model (Positive-ion mode), B) PCA model (Negative-ion mode), C) PLS-DA model (Positive-ion mode), D) PLS-DA model (Negative-ion mode).**

**
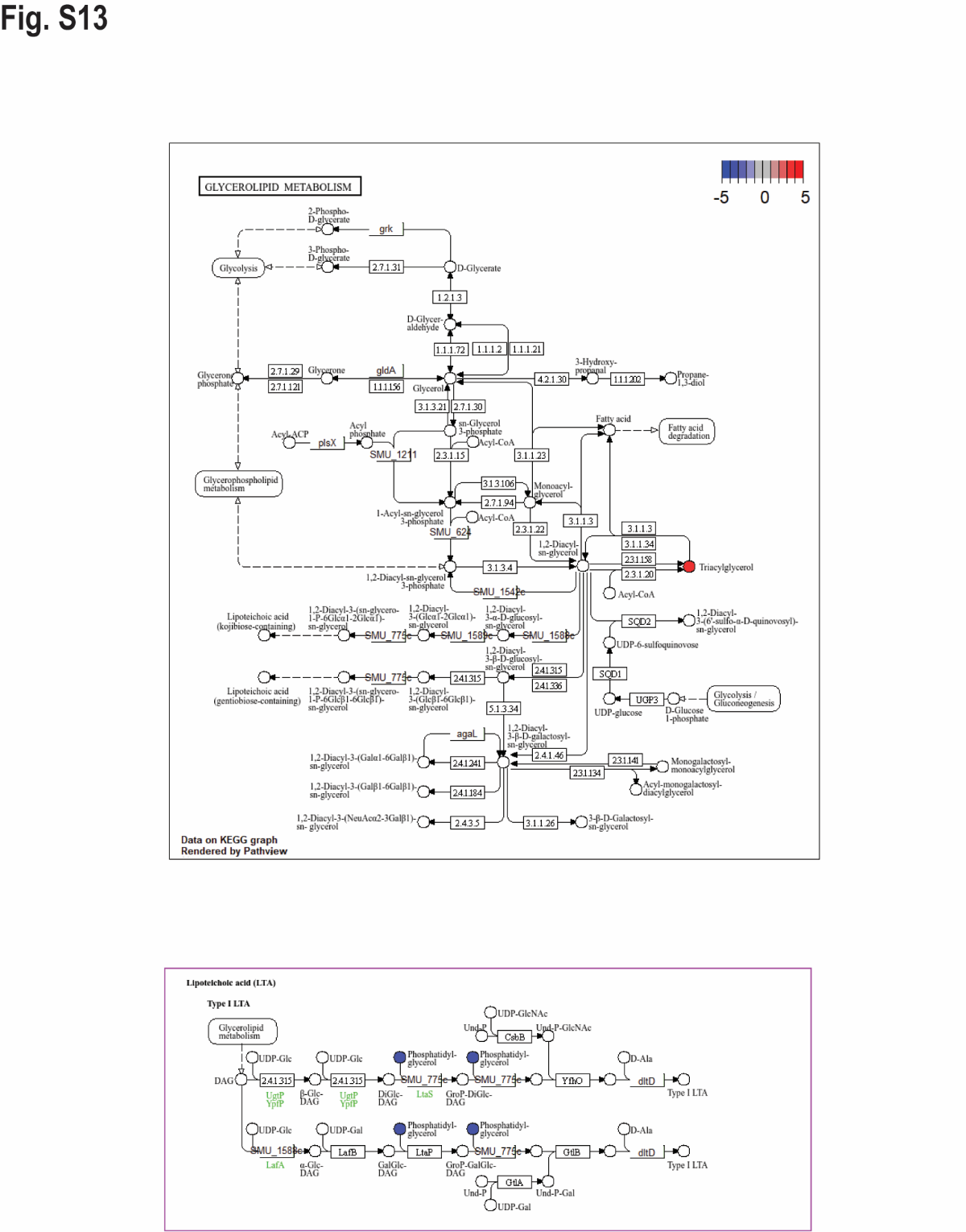
**

**Fig. S13: KEGG pathway heat map of differential lipids (wild-type vs. *ΔhlyX*).**

**References:**

1. Ajdic, D., et al., *Genome sequence of Streptococcus mutans UA159, a cariogenic dental pathogen.* Proc Natl Acad Sci U S A, 2002. **99**(22): p. 14434-9.

2. Kilian, M. and K. Holmgren, *Ecology and nature of immunoglobulin A1 protease-producing streptococci in the human oral cavity and pharynx.* Infect Immun, 1981. **31**(3): p. 868-73.

3. Ge, X. and P. Xu, *Genome-wide gene deletions in Streptococcus sanguinis by high throughput PCR.* J Vis Exp, 2012(69).

4. Biswas, I., J.K. Jha, and N. Fromm, *Shuttle expression plasmids for genetic studies in Streptococcus mutans.* Microbiology (Reading), 2008. **154**(Pt 8): p. 2275-2282.
